# Hydration Ensembles in Proton-Linked Amino-Acid Recognition by Cucurbit[7]uril

**DOI:** 10.1021/acs.jpcb.6c05008

**Published:** 2026-09-09

**Authors:** Ewelina Zaorska, Natasza Jakubik, David A. Rincón, Sivakumar Gurusamy, Maura Malinska

**Affiliations:** Faculty of Chemistry, 49605 University of Warsaw, Pasteura 1, 02-093 Warsaw, Poland

## Abstract

The hydrophobic effect is often considered a major driving force for molecular recognition in water, whether through classical desolvation of nonpolar surfaces or through the release of confined high-energy water from host cavities. However, the interplay between protonation, ion–dipole interactions, and hydration remains poorly understood. Here we show that amino-acid recognition by cucurbit[7]­uril (CB7) is best described as a population-weighted ensemble of proton-linked hydration states, rather than as a single water-release process. By combining pH-dependent isothermal titration calorimetry, X-ray crystallography, molecular dynamics simulations, and interaction-energy analysis, we connect the pH-dependent apparent binding affinities with cavity and portal hydration coupled to ammonium–portal ion–dipole stabilization. Aromatic amino acids differ not by adopting fundamentally different binding modes, but by redistributing population among four hydration states defined by the position of water relative to the cavity and ammonium anchor. Phe and Trp represent opposite limits, with Phe favoring cooperative cavity-water contact and Trp dominated by dry-cavity filling, whereas Tyr and DOPA shift the ensemble toward portal-solvated states. These results show that confined water can strengthen, rather than oppose, hydrophobic recognition when integrated into the ion–dipole binding motif and proton linkage. CB7–amino-acid recognition therefore emerges as a coupled process in which hydrophobic inclusion, ammonium anchoring, hydration, and proton linkage act together.

## Graphical abstract

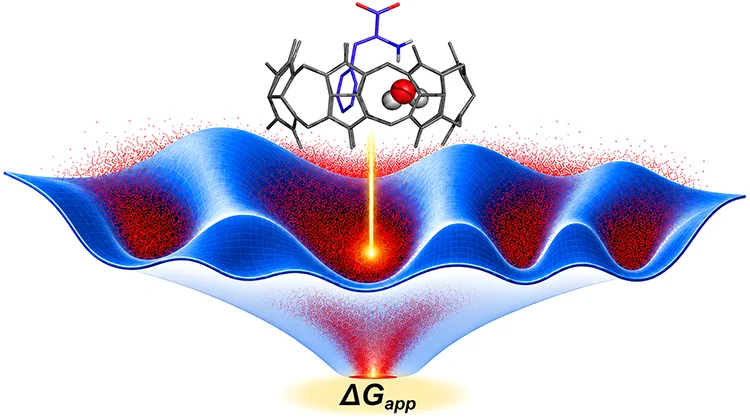

## Introduction

Cucurbit­[7]­uril (CB7) is a macrocyclic host that combines a hydrophobic cavity with two carbonyl-lined portals. This architecture enables simultaneous recognition of nonpolar guest fragments inside the cavity and charged or polar groups at the portals, making CB7 a useful model for molecular recognition in water.^1–7^ Binding in this system reflects a balance of hydrophobic inclusion, ion–dipole anchoring, dispersion, desolvation, hydration rearrangement, and proton linkage.^8–13^ However, although binding free energies are readily measured or computed, they cannot be uniquely decomposed into these physical contributions.

The hydrophobic effect is a central concept in cucurbituril host–guest chemistry and is often associated with the release of confined cavity water.^14–16^ Yet water occupancy alone does not define its thermodynamic role: hydrated binding sites may differ strongly in the free-energy cost of water displacement, and retained water may itself become part of the binding motif.^17–19^ Thus, the relevant question is not simply whether water is present or expelled from the CB7 cavity, but how cavity and portal waters are coupled to guest positioning, protonation, and local stabilization.

Amino acids offer a particularly demanding test case for this coupling. Their side chains can occupy the hydrophobic CB7 cavity, whereas the ammonium and carboxyl groups remain coupled to the carbonyl portals and surrounding solvent. In addition, their protonation states vary with pH, and complex formation can shift these equilibria.^12,13,20–24^ Previous studies have shown that CB7 binds aromatic amino acids with different affinities, with phenylalanine often displaying particularly favorable binding compared with more polar aromatic analogues,^25–28^ and that amino acids can form CB7 complexes with different stoichiometries.^29^ What remains less clear is how side-chain inclusion, cavity hydration, portal hydration, and protonation are coupled in determining the observed binding free energy.

Here, we use CB7 binding to amino acids as a benchmark system to evaluate a population-weighted model of supramolecular recognition in water. In this model, confined water is treated as a state-defining component of the bound complex rather than merely as solvent displaced upon binding. Its spatial distribution within the cavity or at the portals modulates guest orientation, local electrostatic stabilization, and proton linkage. By resolving the bound ensemble into competing hydration states, we show that apparent binding affinities do not arise from a single, uniform water-release event, but emerge from population redistribution among competing proton-linked hydration states.

## Methods

### Crystallization and X-ray Diffraction

Crystals of the CB7 complexes with Phe, Tyr, DOPA, Trp, Met, and Leu (Figures 1 and 2) were obtained from acidic aqueous solutions under the conditions summarized in the Supporting Information (SI, Table S1). Single-crystal X-ray diffraction data were collected at 100 K on an Agilent SuperNova diffractometer equipped with a Cu K*α* microfocus source and either an Atlas or HyPix detector. Data reduction was carried out with CrysAlis PRO. The structures were solved with SHELXT and refined against *F* ^2^ with SHELXL.^30,31^ Full crystallographic data and refinement statistics are given in Tables S2–S3, selected hydrogen-bond geometries in Tables S6–S11, and the geometric descriptors used to compare the CB7–amino-acid binding poses in Tables S4–S5 and Figure S6.

**Figure 1:**
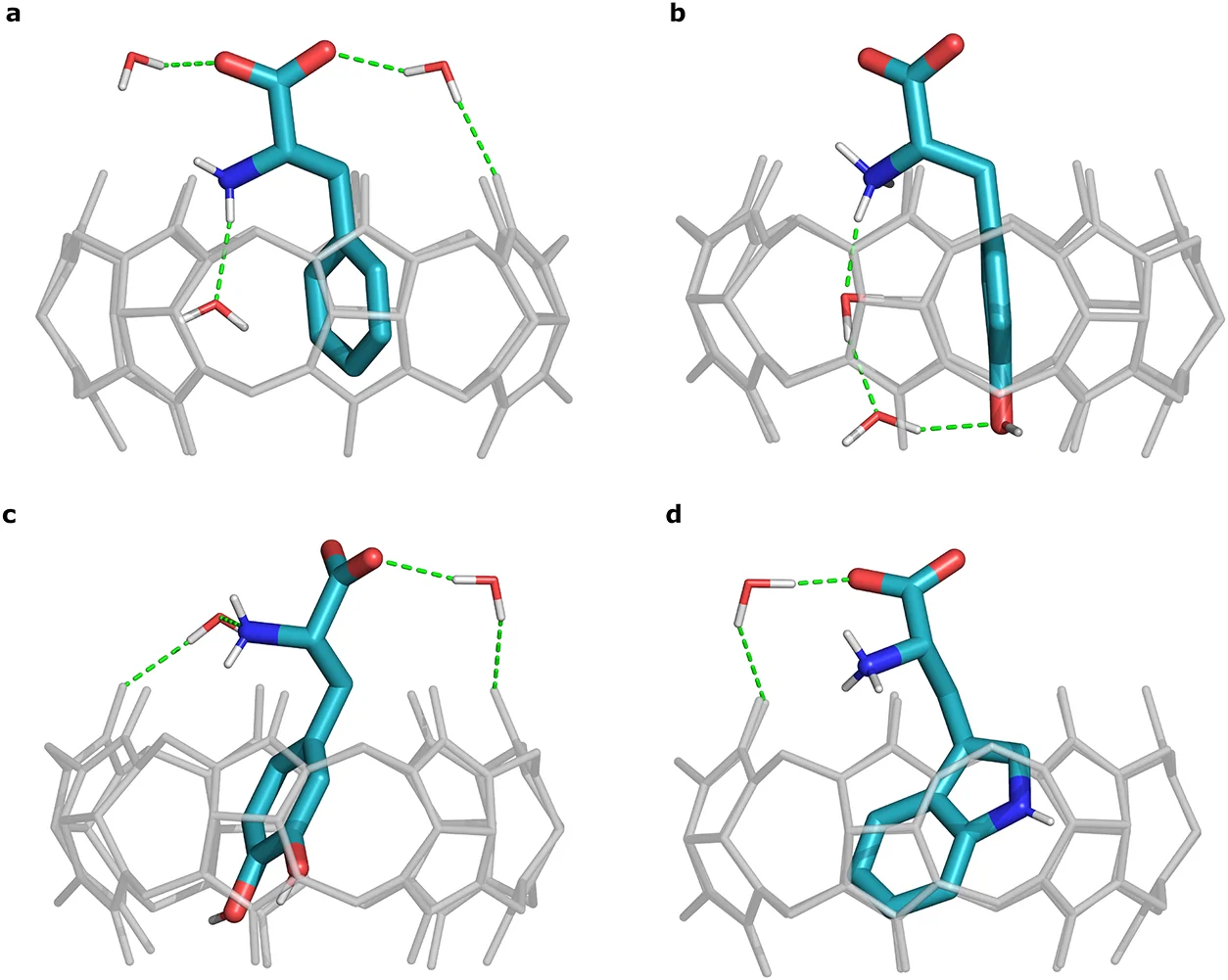
Representative crystal structures of CB7 complexes with aromatic amino acids: (a) Phe, (b) Tyr, (c) DOPA, and (d) Trp. The panels show the binding pose, guest protonation state, and crystallographically observed water molecules. Hydrogen bonds are shown as green dashed lines.

**Figure 2:**
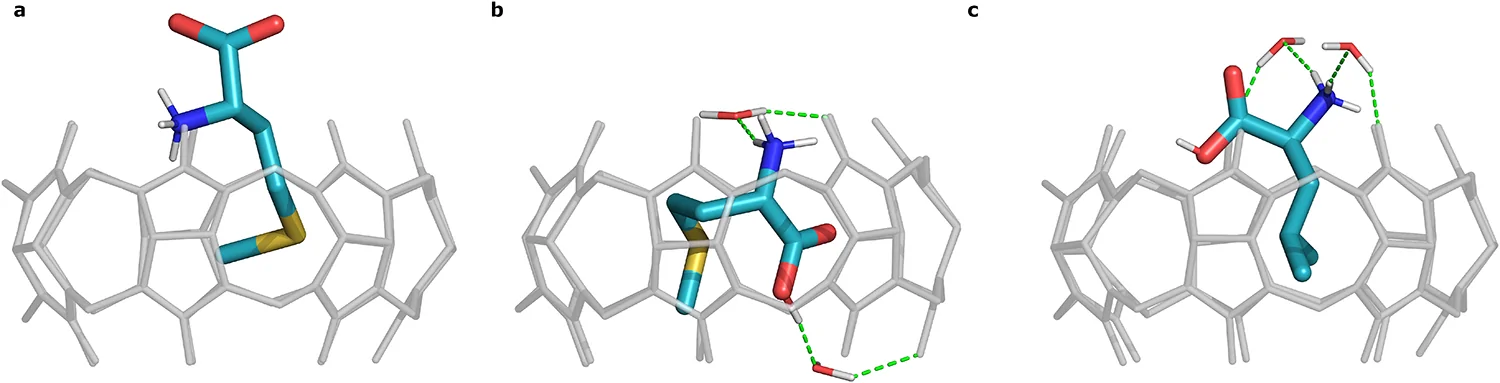
Representative crystal structures illustrating protonation-state diversity in CB7 complexes with aliphatic amino acids: (a) zwitterionic Met, (b) cationic Met, and (c) cationic Leu. Hydrogen bonds to water molecules are shown as green dashed lines.

### Isothermal Titration Calorimetry and Proton-Linkage Analysis

Binding of the amino acids to CB7 was measured by isothermal titration calorimetry using a MicroCal ITC instrument at 25 °C in 20 mM buffer solutions covering the investigated pH range. The same buffer was used for the host and guest solutions in each experiment, and each condition was measured in at least two independent titrations. Apparent dissociation constants were obtained using a 1:1 binding model. Experimental *K*_D_ values and corresponding thermodynamic parameters are summarized in Tables S13–S19.

The pH dependence of the apparent affinity was analyzed using an apparent one-proton linkage model. The fitted parameters were log *K*_D,high_ and p*K*_I,bound_, whereas p*K*_I,free_ was treated as a fixed input parameter. Fits were performed by nonlinear least-squares minimization in log_10_ *K*_D_ space using experimentally propagated uncertainties and a robust soft-L1 loss function. The low-pH limiting dissociation constant was obtained from the fitted parameters. The full thermodynamic linkage scheme, fitting equations, and fitted parameters are provided in SI Sections 4–5 and Table S12.

### Molecular Dynamics Simulations

Molecular dynamics simulations were carried out for the aromatic CB7 complexes with Phe, Tyr, DOPA, and Trp using pmemd.MPI in AMBER22.^32^ CB7 and the amino-acid guests were parameterized with GAFF2, and RESP atomic charges were derived from electrostatic potentials calculated with Gaussian16 at the B3LYP/6-311+G* level. The complexes were solvated explicitly with 2000 TIP3P water molecules under periodic boundary conditions. Production simulations were performed at 298.15 K and 1 bar using a Langevin thermostat and Monte Carlo barostat; long-range electrostatics were treated using particle-mesh Ewald, with a 12 Å cutoff for nonbonded interactions. The simulations of the aromatic complexes were performed for the zwitterionic guest state and therefore characterize hydration-state populations within this protonation state.

For Phe@CB7 and Tyr@CB7, three independent replicas were generated from structures containing an initial cavity water molecule and three additional replicas from cavity-water-free starting structures. The trajectories were analyzed using a common CB7-based geometric reference frame and a mutually exclusive hydration-state classification based on the presence and position of cavity and portal water. The principal states were defined as *core-contact*, *core-no-contact*, *portal-only*, and *dry-cavity*. Full state definitions, trajectory-averaged hydration statistics, replica analyses, and state-resolved geometries are given in SI Section 6, Figures S8–S10, and Tables S20–S39.

### Interaction-Energy Calculations

Quantum-chemical interaction-energy calculations were performed for molecular clusters derived from the crystallographic structures and from representative MD hydration states. Calculations were carried out with ORCA 6.1.^33,34^ XRD-derived clusters were first optimized using r2SCAN-3c^35^ together with the SMD implicit-solvation model for water.^36^ Hydrogen atoms were optimized first with the heavy atoms fixed at their crystallographic positions, followed by optimization of the complete cluster.

Counterpoise-corrected pairwise interaction energies were subsequently calculated on the optimized geometries using ωB97M-V with the def2-TZVP basis set and tight SCF convergence criteria.^37–39^ Basis-set superposition error was corrected using the Boys–Bernardi counterpoise procedure.^40^ The same local interaction-energy framework was applied to representative MD-derived hydration states of Phe@CB7 and Tyr@CB7, and force-field interaction-energy components were additionally evaluated for state-assigned trajectory frames. Optimized structural descriptors and interaction-energy results are reported in SI Tables S40–S43.

## Results and Discussion

### X-ray Structure Analysis

Single-crystal X-ray diffraction revealed inclusion complexes of CB7 with Phe (1), Tyr (2), DOPA (3), Trp (4), Met (5), and Leu (6).^41^ The crystallographic details, including space groups, asymmetric-unit composition, and geometric descriptors, are summarized in the Supporting Information (SI, Tables S2–S11 and Figure S6). Here, we focus on the structural features that define the binding model.

Crystallization was most successful from acidic solution, typically at pH 2–4, where different amino-acid protonation states may be accessible (SI, Table S1). The resulting structures show that CB7 does not impose a single protonation state. Phe, Tyr, DOPA, and Trp are observed as zwitterions (Figure 1), whereas Met contains both zwitterionic and cationic forms within the same crystal, and Leu is observed as a cation (Figure 2). At the same time, all complexes share a conserved binding motif in which the *α*-ammonium group is anchored at one CB7 portal through NH···O contacts with the carbonyl rim (SI, Tables S6–S11). Thus, the ammonium–portal anchor is retained across the series, whereas the guest protonation state, axial position, side-chain placement, and local hydration pattern remain guest-dependent.

The binding poses were compared in a common CB7-based reference frame defined by the two portal centroids and the CB7 mid-plane (SI, Figure S6). This comparison confirms a conserved ammonium–portal anchoring motif, but not a single binding geometry. The ammonium group remains close to the portal plane for Phe, Tyr, and zwitterionic Met, is displaced deeper into the cavity for cationic Met, and is shifted outward for DOPA, Trp, and Leu (SI, Tables S4–S5). The detailed placement of the guest is therefore controlled not only by the ammonium anchor, but also by side-chain structure and protonation state.

The cationic aliphatic complexes illustrate this variability. In cationic Met, the ammonium and carboxylic acid groups are associated with opposite portal regions, with the carboxylic acid contact mediated by a bridge water molecule (Figure 2). In Leu, both polar ends remain associated with the same portal region, and the water-mediated contacts are concentrated near the ammonium-binding side. Thus, cationic protonation changes the portal-binding arrangement and associated hydration network, but does not impose a unique cationic geometry.

These hydration patterns provide the primary structural distinction among the complexes, showing that solvent organization is guest-dependent and forms part of the binding pose itself. Phe contains a single cavity water molecule that hydrogen-bonds directly to the anchored ammonium group, whereas Tyr contains a two-water cavity cluster in which one water molecule maintains a similar ammonium contact. In contrast, no cavity water is observed for DOPA, Trp, Met, or Leu. Several of these structures instead contain external bridge waters that connect the carboxyl/carboxylate group, and in some cases the ammonium region, with the CB7 portals. Thus, the crystal structures identify the retention, exclusion, and redistribution of water as structural variables coupled to guest structure and binding geometry, defining distinct hydration motifs within the CB7 binding pose. These crystal structures provide experimentally observed solid-state snapshots of guest-dependent hydration patterns. Whether these patterns are also recurrent in solution is examined below using MD simulations.

### pH Dependence of Amino Acid Binding to CB7

Binding of amino acids to CB7 cannot be described by a single affinity value independent of solution conditions, because complex formation is coupled to protonation equilibria of the guest (SI, Section 4). We therefore analyzed the pH dependence of binding using a proton-linkage model, in which the measured affinity is treated as an apparent value determined by the population balance between protonation states accessible at a given pH. Throughout the discussion, the terms low- and high-pH limits refer to the asymptotic limits of the fitted proton-linkage model rather than to specific experimental pH values. The fitted *K*_D_ values show a pronounced pH dependence for all studied amino acids (Figure 3); lower *K*_D_ values correspond to stronger apparent binding. The corresponding *K*_D_ and Δ*G* values determined at individual pH values are summarized in the SI (Tables S13–S19).

**Figure 3:**
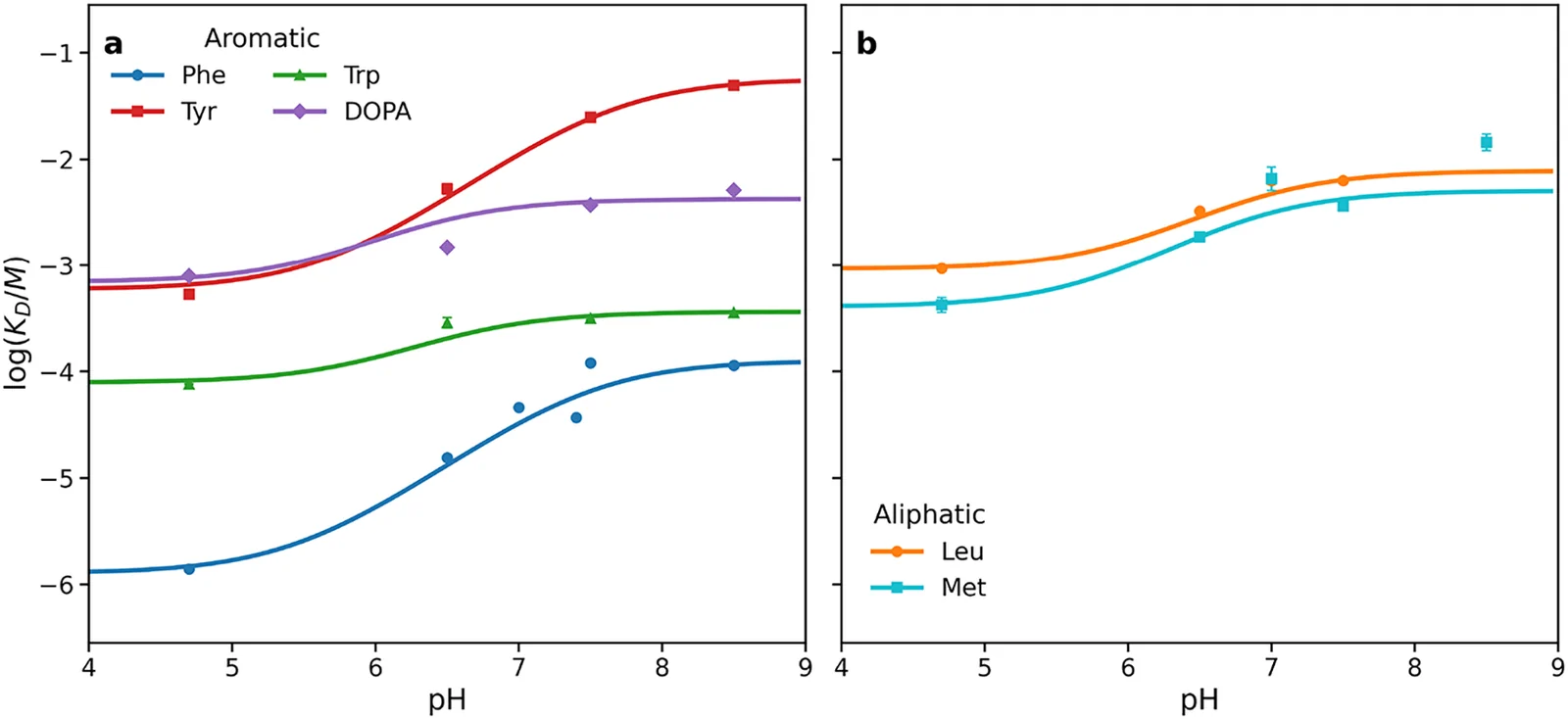
pH dependence of CB7 binding for selected amino acids shown as log­(*K*_D_/M). (a) Aromatic amino acids: Phe, Tyr, Trp, and DOPA. (b) Aliphatic amino acids: Leu and Met. Symbols represent averaged experimental values at each pH, and solid lines show fits to the proton-linkage model. Error bars represent the standard deviation of independent titrations and are smaller than the plotting symbols where not visible.

To compare the free and CB7-bound states, we use the fitted value of the apparent ionization point in the bound state, p*K*_I,bound_. This parameter is the bound-state counterpart of p*K*_I,free_ and describes the pH midpoint of the protonation-linked change in apparent affinity. The difference between p*K*_I,bound_ and p*K*_I,free_ therefore reports how CB7 binding shifts the population balance between protonation states. For all studied amino acids, p*K*_I,bound_ is shifted to higher pH relative to the free amino acid (Table 1), indicating that complex formation stabilizes the more protonated bound state.

**Table 1:** Integrated Summary of pH-Dependent Binding Parameters and Crystallographic Hydration Motifs for CB7–Amino-Acid Complexes^a^

Guest	XRD form	cavity H_2_O	bridge H_2_O	log *K*_D,low‑pHlimit_	log *K*_D,high‑pHlimit_	Δp*K*_I_
Phe	zwitterion	yes	yes	–5.90 ± 0.06	–3.90 ± 0.07	2.00 ± 0.09
Tyr	zwitterion	yes	no	–3.23 ± 0.07	–1.24 ± 0.05	1.98 ± 0.11
DOPA	zwitterion	no	yes	–3.16 ± 0.06	–2.37 ± 0.11	0.78 ± 0.13
Trp	zwitterion	no	yes	–4.10 ± 0.08	–3.44 ± 0.04	0.66 ± 0.09
Met	zwitterion/cation	no	yes	–3.39 ± 0.08	–2.29 ± 0.15	1.10 ± 0.22
Leu	cation	no	yes	–3.03 ± 0.02	–2.11 ± 0.02	0.93 ± 0.03

a Limiting dissociation constants and apparent ionization shifts were obtained from the proton-linkage fit. The low- and high-pH limits are extrapolated limiting values of the fitted model rather than values measured at specific experimental pH points. Reported uncertainties are approximate standard errors obtained from the covariance matrix of the fit; uncertainties of the derived low-pH limiting values were propagated including covariance between the fitted parameters. Structural descriptors refer to the crystallographic complexes.

The limiting affinities and apparent ionization shifts show that proton linkage and binding strength are related but not equivalent descriptors. At the low-pH limit, Phe is by far the strongest binder, followed by Trp, whereas Tyr, DOPA, Met, and Leu show weaker and more comparable affinities. In contrast, the apparent protonation shifts follow three approximate regimes: Phe and Tyr show the largest shifts, Met and Leu are intermediate, and Trp and DOPA show the smallest shifts. This decoupling shows that neither aromatic inclusion nor the conserved ammonium–portal contact is sufficient to explain the pH profiles. Instead, each profile reflects how protonation is coupled to the specific bound-state environment of a given guest.

### MD Evidence for Hydration-State Populations

The crystal structures revealed guest-dependent differences in protonation state, ammonium position, and local solvent organization, but they provide only static snapshots of selected solid-state arrangements. We therefore used MD simulations to determine whether the crystallographically observed hydration motifs correspond to recurrent bound-state hydration patterns in solution, or whether they represent transient configurations within a broader hydration landscape. The MD simulations of the aromatic complexes were performed for the zwitterionic guest state, which is the predominant protonation state over the central pH range considered here; they therefore resolve hydration-state populations within this protonation state rather than explicitly simulating pH-dependent protonation equilibria.

In particular, we focused on water molecules located inside the CB7 cavity and in the portal regions, because this hydration pattern most clearly differentiates the Phe and Tyr crystal structures from those of DOPA, Trp, Met, and Leu. Each MD frame was assigned to one of four mutually exclusive hydration states according to the position of water relative to the CB7 cavity and its direct contact with the ammonium group: *core-contact*, in which a cavity water molecule directly interacts with the ammonium group; *core-no-contact*, in which cavity water is present but does not form a direct ammonium contact; *portal-only*, in which water is located near the portal region but no cavity water is present; and *dry-cavity*, in which neither cavity water nor portal water is present within the analyzed regions.

When applied to the trajectories, this classification shows how the crystallographic hydration patterns are expressed within the solution-state ensemble. For Phe and Tyr, the simulations recover a distinct *core-contact* state, whereas DOPA is dominated by *portal-only* hydration and Trp predominantly samples the *dry-cavity* state (Figure 4). The XRD/MD water-density overlay further shows that the crystallographic cavity waters in Phe and Tyr coincide with preferred hydration regions sampled in solution (Figure 5).

**Figure 4:**
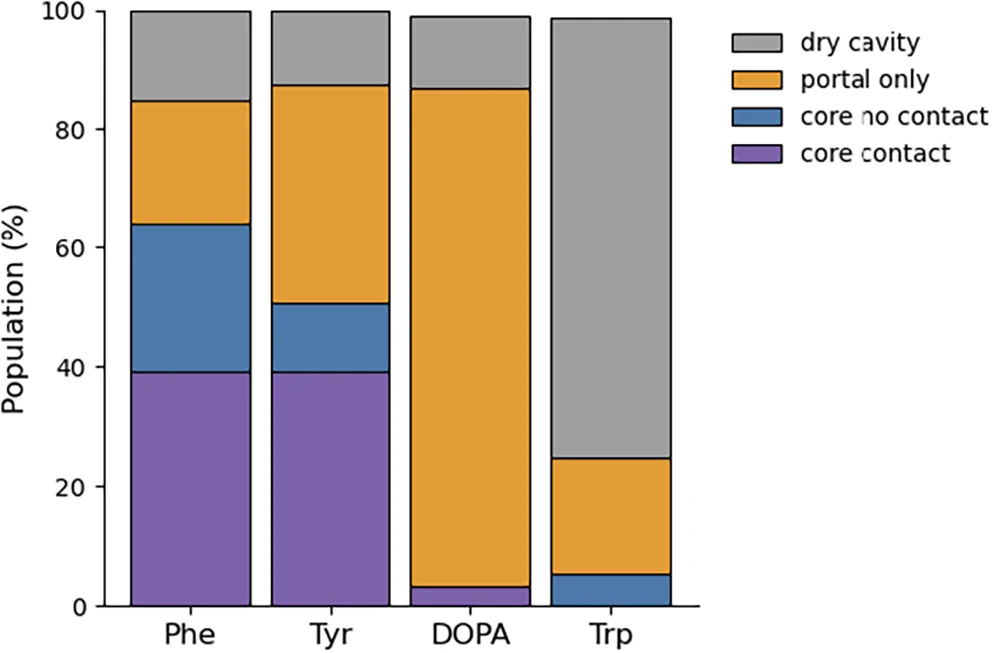
Population of MD-derived hydration states for the aromatic amino acid complexes with CB7. The relative populations of the four states—*core-contact*, *core-no-contact*, *portal-only*, and *dry-cavity*—are shown for Phe, Tyr, DOPA, and Trp. The reported populations were obtained from MD simulations of the zwitterionic complexes and describe the hydration-state distributions within this protonation state. The distributions reveal clear guest-dependent preferences, including substantial *core-contact* populations for Phe and Tyr, a dominant *portal-only* state for DOPA, and a dominant *dry-cavity* state for Trp. Percentages may not sum exactly to 100% due to omission of minor states and rounding.

**Figure 5:**
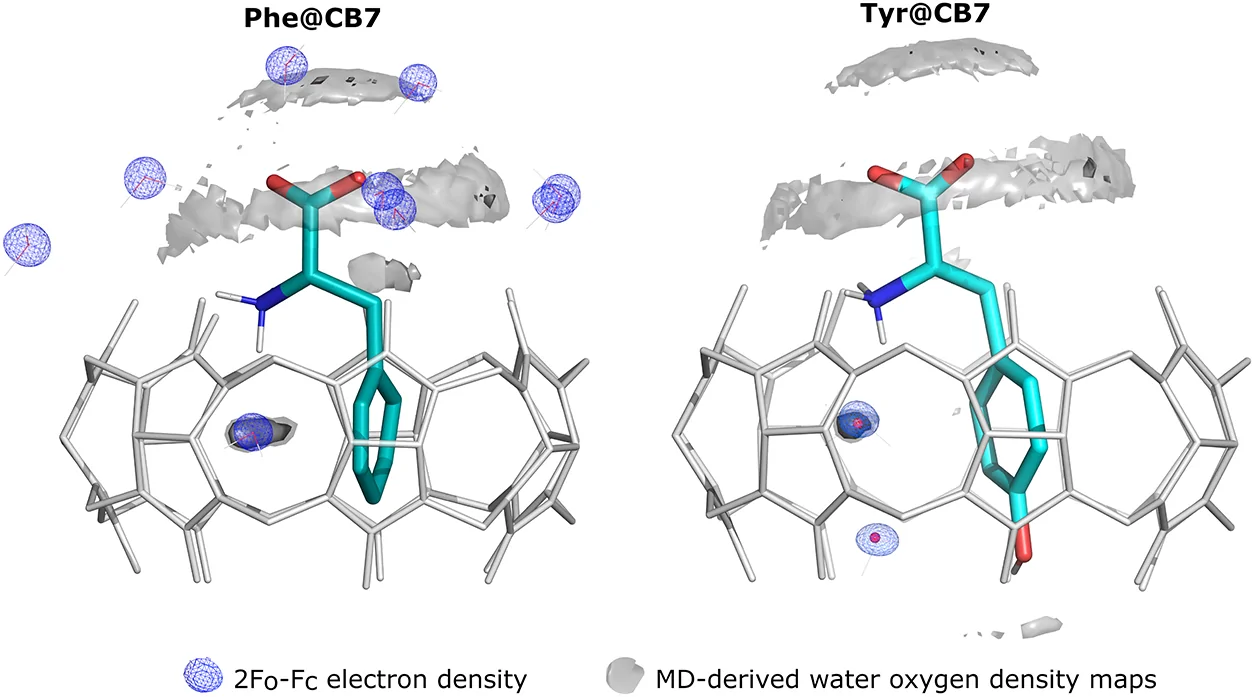
Overlay of crystallographic and MD-derived hydration patterns for Phe@CB7 and Tyr@CB7. Crystallographic 2Fo–Fc electron density around selected water molecules is shown as blue mesh and contoured at 2.5 e Å^–3^. MD-derived water oxygen density maps are shown as grey surfaces. The comparison highlights similar preferred hydration regions in the CB7 cavity and around the carboxylate/portal region.

Although the *core-contact* motif is available to both Phe and Tyr, the two complexes differ in the population balance between hydration states. Phe maintains a substantial *core-contact* population and shows comparatively small geometric changes between the hydration states, consistent with a robust inclusion geometry. Tyr can form the same *core-contact* motif, but the *core-contact* and *portal-only* states are more closely balanced than in Phe, indicating stronger competition between cavity hydration and portal-associated solvation. Thus, the key difference between Phe and Tyr is not the existence of a productive *core-contact* state, but its population within the ensemble (SI, Tables S20–S33).

The *core-contact* state is structurally distinct from the other hydration states. In Phe@CB7 and Tyr@CB7, direct contact between cavity water and the ammonium group is coupled to a more deeply inserted guest geometry and favors an upright aromatic-ring orientation, closely matching the crystallographic structures (Figure 6). The retained cavity water therefore complements the ammonium–portal interaction rather than replacing it (SI, Figures S8–S10). Importantly, this change is not localized solely within the CB7 cavity. The *core-contact* geometry also alters the position of the carboxylate terminus relative to the portal region, thereby changing the accessibility of external bridge-water contacts. When the direct cavity-water/ammonium contact is absent, the guest samples geometries in which the carboxylate group is more exposed to portal hydration and the bridge-water motif becomes more accessible. Thus, the MD-derived states describe a redistribution of hydration across the whole CB7–amino-acid interface, rather than the simple presence or absence of a water molecule in the cavity.

**Figure 6:**
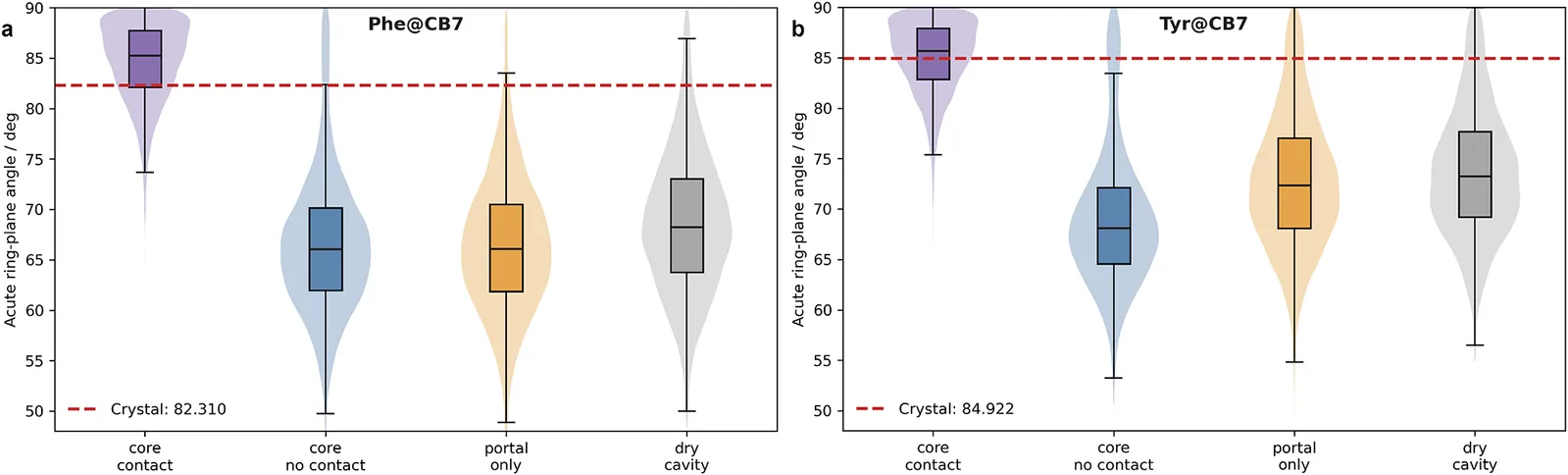
State-resolved distributions of representative geometrical descriptor for Phe@CB7 and Tyr@CB7 from MD simulations. The four hydration states are shown as violin plots with embedded box plots, and optimized XRD-derived reference geometries are indicated by red dashed lines. For Tyr@CB7, the three crystallographically independent occupied complexes converge to the same *core-contact* reference minimum upon optimization; therefore, a single optimized reference value is shown. (a, b) Acute ring-plane angle for Phe@CB7 and Tyr@CB7, respectively. In both systems, the optimized XRD-derived reference geometry is closest to the *core-contact* state. Tyr adopts higher ligand positions and exhibits a more distinct *portal-only* geometry than Phe.

Residence-event analysis further shows that this hydration site is dynamically maintained: individual water molecules exchange on the simulation time scale, but the cavity region remains frequently occupied in Phe@CB7 and Tyr@CB7 (SI, Table S21). The crystallographic waters should therefore be interpreted as markers of preferred hydration regions rather than immobile solvent positions.

DOPA and Trp define two contrasting limits of the same hydration landscape. DOPA is dominated by *portal-only* hydration, whereas Trp predominantly samples the *dry-cavity* state and also shows reduced portal and bridge-water hydration. Trp therefore represents a relatively dehydrated binding interface, not merely a cavity-dehydrated state. These trends show that aromatic inclusion alone does not determine the hydration pattern.

### Interaction-Energy Analysis

The interaction-energy analysis places the crystallographic and MD-derived binding motifs on a local energetic scale, separating direct CB7–amino-acid stabilization from water-mediated contacts within the same structural environment. In the XRD-derived clusters, the direct CB7···amino-acid interaction primarily reflects the expected sensitivity to guest protonation state. When the relevant crystallographic water contacts are included, the zwitterionic aromatic complexes show large stabilizing local interaction energies, approaching or exceeding −200 kJ mol^–1^ (SI, Table S41). In contrast, zwitterionic Met shows only about half of this stabilization, consistent with its weaker local portal stabilization in this binding arrangement. The cationic Met and Leu complexes display much stronger direct CB7···amino-acid interaction energies, showing that guest protonation strongly enhances local electrostatic stabilization at the CB7 portal. This protonation-dependent baseline provides the reference against which the role of local hydration can be assessed.

The pairwise water-contact energies then show that water is not a peripheral contribution to these motifs, but part of their local stabilization. In Phe and Tyr, the cavity contact waters stabilize the ammonium region and contribute to the *core-contact* motif. In DOPA, favorable direct CB7–amino-acid stabilization instead coexists with bridge-water contacts at the carboxylate and portal region, illustrating that the absence of cavity water does not imply loss of host–guest stabilization. In Trp, Met, and Leu, bridge waters connect the carboxylate or carboxylic acid group, and in some cases also the ammonium group, with the CB7 portal. These water-mediated contacts provide additional local stabilization, with amino-acid–water interaction energies reaching several tens of kJ mol^–1^ (SI, Table S41). Although these pairwise energies are not directly comparable to hydration free energies or to the enthalpy of cavity-water release, their magnitude shows that local water contacts can make a substantial contribution to selected binding geometries.

To complement the analysis of XRD-derived clusters, representative geometries were also selected from the MD-defined hydration states of Phe@CB7 and Tyr@CB7 (SI, Tables S42–S43). These representative clusters were chosen to capture geometrically typical configurations of each hydration state, whereas their optimized structures identify locally favorable realizations of these motifs. The comparison with state-resolved force-field interaction energies shows that the same hydration states are dynamically broadened, with fluctuating water positions and contact geometries. Thus, an optimized representative cluster can describe a deep local minimum within a given hydration state, while the state itself remains distributed over a wider range of related configurations.

The state-resolved interaction energies show that the strongest direct CB7–amino-acid interaction is not necessarily associated with the most stabilizing amino-acid–water contact. For both Phe and Tyr, the *core-contact* state contains a strongly stabilizing amino-acid–contact-water interaction, whereas the direct CB7–amino-acid term is more stabilizing in the *core-no-contact* and *dry-cavity* states. This is particularly important for Tyr@CB7: residence-event analysis shows that individual cavity waters exchange on the simulation time scale, but the *core-contact* hydration site remains recurrent (SI, Table S21). Thus, the relevant stabilizing motif is not a permanently bound water molecule, but a repeatedly occupied hydration site that supports the ammonium-contact geometry when populated. The stabilization of a hydration state therefore cannot be rationalized from the direct CB7–amino-acid interaction alone, but reflects a balance of host–guest, guest–water, host–water, and water–water interactions across a dynamic ensemble. In this sense, cavity and portal water molecules actively modulate the local stabilization of CB7–amino-acid complexes, rather than acting as passive spectators.

### Integrated Binding-State Model

The CB7–amino-acid complexes are best described as ensembles of proton-linked bound states, in which cavity filling, ammonium–portal anchoring, and local water organization are coupled in a guest-dependent manner. This model integrates the crystallographic hydration motifs, the MD-derived state populations, the pH-dependent apparent affinities, and the local interaction-energy analysis into a single binding-state description.

The crystal structures provide experimentally observed snapshots of this landscape, whereas MD simulations reveal the corresponding hydration states and their populations (Figures 4–6 and SI, Tables S20–S39). For Phe and Tyr, the crystallographic cavity-water motif corresponds to a recurrent *core-contact* state in solution. This state is coupled not only to water occupancy, but also to ligand position, aromatic-ring orientation, direct water–ammonium contact, and preservation of the ammonium–portal anchor. Thus, the crystallographic waters mark preferred hydration regions that are sampled dynamically rather than fixed solvent positions.

The bound ensembles differ mainly in how population is distributed among these hydration motifs. Phe and Trp define two opposite limits among the aromatic guests: Phe favors a productive *core-contact* motif involving cavity water, the ammonium group, and the portal anchor, whereas Trp follows a predominantly *dry-cavity* filling mode with reduced local hydration. Tyr and DOPA occupy intermediate or redistributed regimes. Tyr accesses the same *core-contact* motif as Phe, but the population of this state is more closely balanced with those of the *portal-only* and *core-no-contact* states. DOPA, in contrast, shifts strongly toward portal-associated hydration, with only marginal population of the *core-contact* state. Thus, the aromatic series does not represent a sequence of unrelated binding modes, but a redistribution of population across a common hydration-state landscape.

The aliphatic guests extend this model to non-aromatic, portal-stabilized binding. Although no cavity water is observed in the Met and Leu crystal structures, both guests show intermediate apparent protonation shifts, larger than those of DOPA and Trp but smaller than those of Phe and Tyr. This behavior is consistent with proton linkage amplified through hydrated cationic portal arrangements, where water-mediated contacts at the carbonyl rim stabilize portal-bound cationic motifs rather than a *core-contact* cavity-water state (SI, Table S41).

This model provides a structural interpretation of the pH-dependent affinity trends (Figure 3 and Table 1). At the low-pH limit of the proton-linkage model, Phe and Trp are the strongest binders and define two contrasting limits of the hydration landscape: Phe is enriched in a water-retaining *core-contact* state, whereas Trp is dominated by the *dry-cavity* state, representing the water-release limit of the same hydration landscape. Tyr and DOPA occupy more redistributed hydration regimes, with stronger competition from portal-associated states, whereas Met and Leu extend the same binding-state picture toward hydrated, portal-stabilized cationic arrangements. The affinity trends can therefore be understood in terms of how each guest distributes stabilization across cavity filling, ammonium–portal anchoring, and local hydration. At the high-pH limit, the apparent affinity is reduced as the proton-linked population balance shifts away from the more protonated bound state, with the magnitude of this response depending on how strongly each complex relies on ammonium–portal contacts, ammonium–water interactions, and the associated hydration network. Thus, the pH response emerges from guest-dependent redistribution within a common proton-linked binding landscape rather than from a single universal hydration mechanism.

## Conclusions

By combining crystallography, pH-dependent thermodynamics, MD simulations, and interaction-energy analysis, we show that amino-acid recognition by CB7 cannot be reduced to a single hydrophobic inclusion mechanism or a universal binding geometry. The measured affinities are apparent quantities shaped by proton linkage, and the bound complexes are best described as population-weighted ensembles in which cavity filling, ammonium–portal anchoring, protonation equilibria, and local water organization are coupled rather than separable. Water is therefore not merely released upon binding, but dynamically recaptured within these complexes, acting as a guest-dependent stabilizing element that either reinforces the ammonium–portal anchor through cavity contact or supports alternative portal-associated hydration networks. In this way, water helps determine which bound states are populated, and thereby shapes the apparent thermodynamic response of CB7 recognition in water.

## Supplementary Material















## Notes

Crystal structure of the Trp@CB7 complex (CCDC 2571852) (CIF)
Crystal structure of the Leu@CB7 complex (CCDC 2571853) (CIF)
Crystal structure of the DOPA@CB7 complex (CCDC 2571854) (CIF)
Crystal structure of the Met@CB7 complex (CCDC 2571855) (CIF)
Crystal structure of the Tyr@CB7 complex (CCDC 2571856) (CIF)
Crystal structure of the Phe@CB7 complex (CCDC 2571857) (CIF)
Experimental procedures; crystallographic methods and structure refinement; geometric analysis of CB7–amino acid complexes; proton-linkage model and derivation of the apparent binding equations; ITC measurements and fitting procedures; molecular dynamics simulation protocols; hydration-state analysis; water occupancy and residence-time analyses; state-resolved structural and interaction-energy analyses; thermodynamic linkage scheme describing CB7 binding coupled to the protonation equilibria of the amino acid; state-resolved distributions of acute ring-plane angle for aromatic amino acid complexes with CB7 obtained from MD simulations; water occupancy and residence-event analysis for the core and portal hydration regions in Phe@CB7 and Tyr@CB7, and bridge-summary analysis for the Phe@CB7 replicas initiated without a cavity water molecule (PDF)
