# Supplementary material for: Hydration Ensembles in Proton-Linked Amino-Acid Recognition by Cucurbit[7]uril
Source: J Phys Chem B. 2026 Sep 9;130(41):10961–9. doi: 10.1021/acs.jpcb.6c05008 (PMC13679039; doi:10.1021/acs.jpcb.6c05008)
Supplement: Supplementary file 1 [file jp6c05008_si_007.pdf]

Supporting Information

Hydration Ensembles in Proton-Linked Amino-Acid Recognition by Cucurbit[7]uril

Ewelina Zaorska, Natasza Jakubik, David A. Rincón, Sivakumar Gurusamy, Maura Malinska*

Faculty of Chemistry, University of Warsaw, Pasteura 1, 02-093 Warsaw, Poland
mmalinska@chem.uw.edu.pl

Contents

1 Materials and buffer preparation for ITC **S8**

2 Crystallization **S8**

3 X-ray diffraction **S9**

4 Protonation linkage of binding affinities **S20**

5 ITC measurements **S21**

5.1 pH-dependent ITC fitting procedure S22

6 Molecular Dynamics Simulations **S25**

7 Interaction Energy Calculations **S34**

List of Figures

S1 General overview of the binding pose of Phe (charge 0) (a), Tyr (charge 0) (b), DOPA (charge 0) (c), Trp (charge 0) (d), Met (charge 0) (e), Met (charge +1) (f), Leu (charge +1) (g) in the CB7 cavity. The CB7 molecule is depicted using a stick representation, and the van der Waals surfaces are shown. The CB7 molecule is clipped to show the amino acid position. Hydrogen bonds are represented as red dashed lines. S9

S2 Phe (a) and Tyr (b) within the CB7 cavity, highlighting water molecules from the crystal structure **1** and **2**, respectively. Heavy atoms are displayed with anisotropic displacement parameters (ADPs), while only the positions of polar hydrogen atoms are shown. Labels indicate the positions of hydrogen bond donors and acceptors. S12

S3 DOPA (a) and Trp (b) within the CB7 cavity, highlighting water molecules from the crystal structure **3** and **4**, respectively. Heavy atoms are displayed with anisotropic displacement parameters (ADPs), while only the positions of polar hydrogen atoms are shown. Labels indicate the positions of hydrogen bond donors and acceptors. S13

S4 Met in zwitterionic form (a) and cationic form (b) within the CB7 cavity, highlighting water molecules from the crystal structure **5**. Heavy atoms are displayed with ADPs, while only the positions of polar hydrogen atoms are shown. Labels indicate the positions of hydrogen bond donors and acceptors. S13

S5 Leu in cationic form within the CB7 cavity, highlighting water molecules from the crystal structure **6**. Heavy atoms are displayed with anisotropic displacement parameters (ADPs), while only the positions of polar hydrogen atoms are shown. Labels indicate the positions of hydrogen bond donors and acceptors. S14

S6 Definition of the geometric descriptors used in the analysis of CB7–amino acid complexes. (a) The CB7 reference frame is defined by the centroids of portal 1, portal 2, and the CB7 mid-plane; the portal1→portal2 vector defines the axial coordinate. (b) The projected positions of N_α and the ligand center of geometry (COG) are measured along this axis from portal 1. (c) For aromatic guests, the acute ring-plane angle is defined as the acute angle between the aromatic ring plane and the CB7 mid-plane. S15

S7 Thermodynamic linkage scheme describing CB7 binding coupled to the protonation equilibria of the amino acid. S20

S8 State-resolved distributions of N_α axial position for aromatic amino acid complexes with CB7 obtained from MD simulations. The four hydration states are shown as violin plots with embedded box plots, and the corresponding optimized XRD-derived reference values are indicated by red dashed lines. (a) Phe@CB7, (b) Tyr@CB7, (c) DOPA@CB7, and (d) Trp@CB7. State populations are given below the x-axis labels. S26
S9 State-resolved distributions of ligand COG axial position for aromatic amino acid complexes with CB7 obtained from MD simulations. The four hydration states are shown as violin plots with embedded box plots, and the corresponding optimized XRD-derived reference values are indicated by red dashed lines. (a) Phe@CB7, (b) Tyr@CB7, (c) DOPA@CB7, and (d) Trp@CB7. State populations are given below the x-axis labels. S27
S10 State-resolved distributions of acute ring-plane angle for aromatic amino acid complexes with CB7 obtained from MD simulations. The four hydration states are shown as violin plots with embedded box plots, and the corresponding optimized XRD-derived reference values are indicated by red dashed lines. (a) Phe@CB7, (b) Tyr@CB7, (c) DOPA@CB7, and (d) Trp@CB7. State populations are given below the x-axis labels. S28

List of Tables

S1 Summary of crystallization conditions. CB7 0.007 mmol, 1 equiv. S8
S2 Experimental details for structures **1**–**3**. Experiments were carried out at 100 K with Cu Kα radiation. S10
S3 Experimental details for structures **4**–**6**. Experiments were carried out at 100 K with Cu Kα radiation. S11
S4 Geometric parameters describing the orientation of the amino acid backbone relative to the CB7 host in crystal structures **1**–**6**. The geometry is characterized by the tilt of the ammonium group, defined by the angle Portal 1–CB7 midpoint–N_α, the tilt of the carboxyl/carboxylate group, defined by the angle Portal 1–CB7 midpoint–C, and the tilt of the α-carbon, defined by the angle Portal 1–CB7 midpoint–C_α. Atom labels are retained to distinguish symmetry-independent complexes in the asymmetric unit. S15
S5 Geometric parameters for each ligand position in crystal structures **1**–**6**, describing the binding geometry relative to portal 1 (P1). The first parameter is the signed perpendicular distance between the nitrogen atom of the amine group and the average plane of portal 1, denoted as $d(\mathrm{P1} \ldots \mathrm{N})$. Negative values indicate that the atom is located above the portal plane, that is, farther from the CB7 midpoint, whereas positive values indicate that it lies below the portal plane, toward the CB7 interior. The second parameter is the analogous signed perpendicular distance for the carboxyl carbon atom, $d(\mathrm{P1} \ldots \mathrm{C})$. The third parameter is the angle between the P1 plane and the average plane of the aromatic ring. For amino acids without aromatic side chains, this parameter is not defined. All geometric parameters in this table were measured in Mercury from the crystallographic model. S16
S6 Selected Hydrogen-Bond Parameters for the Crystal Structure of the Complex Between CB7 and Phe (**1**) S17
S7 Selected Hydrogen-Bond Parameters for the Crystal Structure of the Complex Between CB7 and Tyr (**2**) S18
S8 Selected Hydrogen-Bond Parameters for the Crystal Structure of the Complex Between CB7 and DOPA (**3**) S18

S9 Selected Hydrogen-Bond Parameters for the Crystal Structure of the Complex Between CB7 and Trp (**4**) . . . S18
S10 Selected Hydrogen-Bond Parameters for the Crystal Structure of the Complex Between CB7 and Met (**5**) . . . S19
S11 Selected Hydrogen-Bond Parameters for the Crystal Structure of the Complex Between CB7 and Leu (**6**) . . . S19
S12 Parameters obtained from pH-dependent ITC fitting for CB7 complexes with amino acids. Dissociation constants are given in mM. $K_{D,\text{low}}$ and $K_{D,\text{high}}$ denote the low- and high-pH limiting values extrapolated from the fitted linkage model rather than values measured at specific experimental pH points. Reported uncertainties are approximate standard errors obtained from the covariance matrix of the fit; uncertainties of the derived low-pH limiting values were propagated including covariance between the fitted parameters. . . . S22
S13 Observed pH-dependent ITC dissociation constants used for the fitting. Values of $K_{D,\text{obs}}$ are given as mean ± SD in mM; n is the number of independent fitted titrations retained after data cleaning. The corresponding observed Gibbs free energies were calculated from the mean $K_{D,\text{obs}}$ values using $\Delta G_\text{obs} = RT \ln K_{D,\text{obs}}$, with K_D expressed in mol L^{-1}. . . . S23
S14 ITC-derived binding parameters for the complexation of Phe with CB7 at different pH values. . . . S23
S15 ITC-derived binding parameters for the complexation of Tyr with CB7 at different pH values. . . . S24
S16 Observed pH-dependent ITC dissociation constants for the complexation of DOPA with CB7. Values of $K_{D,\text{obs}}$ are given as mean ± SD in mM; n is the number of independent fitted titrations retained after data cleaning. The corresponding observed Gibbs free energies were calculated from the mean $K_{D,\text{obs}}$ values using $\Delta G_\text{obs} = RT \ln K_{D,\text{obs}}$, with K_D expressed in mol L^{-1}. . . . S24
S17 Observed pH-dependent ITC dissociation constants for the complexation of L-Trp with CB7. Values of $K_{D,\text{obs}}$ are given as mean ± SD in mM; n is the number of independent fitted titrations retained after data cleaning. The corresponding observed Gibbs free energies were calculated from the mean $K_{D,\text{obs}}$ values using $\Delta G_\text{obs} = RT \ln K_{D,\text{obs}}$, with K_D expressed in mol L^{-1}. . . . S24
S18 Observed pH-dependent ITC dissociation constants for the complexation of Met with CB7. Values of $K_{D,\text{obs}}$ are given as mean ± SD in mM; n is the number of independent fitted titrations retained after data cleaning. The corresponding observed Gibbs free energies were calculated from the mean $K_{D,\text{obs}}$ values using $\Delta G_\text{obs} = RT \ln K_{D,\text{obs}}$, with K_D expressed in mol L^{-1}. . . . S24
S19 Observed pH-dependent ITC dissociation constants for the complexation of L-Leu with CB7. Values of $K_{D,\text{obs}}$ are given as mean ± SD in mM; n is the number of independent fitted titrations retained after data cleaning. The corresponding observed Gibbs free energies were calculated from the mean $K_{D,\text{obs}}$ values using $\Delta G_\text{obs} = RT \ln K_{D,\text{obs}}$, with K_D expressed in mol L^{-1}. . . . S25
S20 Trajectory-averaged water statistics for the amino acid@CB7 replicas. Reported values were calculated over all analysed frames and summarize the average hydration of the core, portal, and bridge regions. . . . S27
S21 Water occupancy and residence-event analysis for the core and portal hydration regions in Phe@CB7 and Tyr@CB7. Occ. is the percentage of analysed MD frames in which at least one water oxygen atom is present in the region. N_ev is the number of continuous residence events identified from the per-frame hydration assignment. Residence times are given as median, mean, and maximum values. Saved trajectory frames were spaced by 10 ps. . . . S28

S22 Summary of the full 300 ns MD analyses for the Phe@CB7 replicas initiated with a cavity water molecule (HOH) and without cavity water (nHOH). The table compares the mean axial position of the ammonium nitrogen (N_α), mean axial position of the ligand center of geometry (COG), mean acute angle between the aromatic ring plane and the CB7 mid-plane, and the populations of the major water/positioning states identified in the trajectory analysis. S29

S23 State-resolved geometry for the Phe@CB7 replicas initiated with a cavity water molecule (HOH). Only the major MD-derived hydration states are shown: *core-contact*, *core-no-contact*, *portal-only*, and *dry-cavity*, as defined in the Molecular Dynamics section. Reported values are mean ± standard deviation calculated over frames assigned to a given state. Low-population states were omitted for clarity. S29

S24 State-resolved geometry for the Phe@CB7 replicas initiated without a cavity water molecule (nHOH). Only the major MD-derived hydration states are shown: *core-contact*, *core-no-contact*, *portal-only*, and *dry-cavity*, as defined in the Molecular Dynamics section. Reported values are mean ± standard deviation calculated over frames assigned to a given state. Low-population states were omitted for clarity. S29

S25 Bridge-summary analysis for the Phe@CB7 replicas initiated with a cavity water molecule (HOH). Only the major MD-derived hydration states are shown: *core-contact*, *core-no-contact*, *portal-only*, and *dry-cavity*, as defined in the Molecular Dynamics section. For each state, the table reports the number of frames, population, mean total number of bridge waters, fraction of frames containing at least one bridge water, mean numbers of UU and UV hydrogen bonds, mean number of core waters, and mean number of core-contact waters. Low-population states were omitted for clarity. S30

S26 Bridge-summary analysis for the Phe@CB7 replicas initiated without a cavity water molecule (nHOH). Only the major states are shown: *core-contact*, *core-no-contact*, *portal-only*, and *dry-cavity*, as defined in the Molecular Dynamics section. For each state, the table reports the number of frames, population, mean total number of bridge waters, fraction of frames containing at least one bridge water, mean numbers of UU and UV hydrogen bonds, mean number of core waters, and mean number of core-contact waters. Low-population states were omitted for clarity. S30

S27 Per-frame water statistics for the Phe@CB7 trajectories. Reported values were calculated over the full 300 ns trajectories (30 000 analysed frames per replica). The table summarizes the mean numbers of core and portal waters, the mean number of core-contact waters, and the fractions of frames containing core or portal waters. S30

S28 Summary of the full 300 ns MD analyses for the Tyr@CB7 replicas initiated with a cavity water molecule (HOH) and without cavity water (nHOH). The table compares the mean axial position of the ammonium nitrogen (N_α), mean axial position of the ligand center of geometry (COG), mean acute angle between the aromatic ring plane and the CB7 mid-plane, and the populations of the major water/positioning states identified in the trajectory analysis. S30

S29 State-resolved geometry for the Tyr@CB7 replicas initiated with a cavity water molecule (HOH). Only the major MD-derived hydration states are shown: *core-contact*, *core-no-contact*, *portal-only*, and *dry-cavity*, as defined in the Molecular Dynamics section. Reported values are mean ± standard deviation calculated over frames assigned to a given state. Low-population states were omitted for clarity. S31

S30 State-resolved geometry for the Tyr@CB7 replicas initiated without a cavity water molecule (nHOH). Only the major MD-derived hydration states are shown: *core-contact*, *core-no-contact*, *portal-only*, and *dry-cavity*, as defined in the Molecular Dynamics section. Reported values are mean ± standard deviation calculated over frames assigned to a given state. Low-population states were omitted for clarity. S31

S31 Bridge-summary analysis for the Tyr@CB7 replicas initiated with a cavity water molecule (HOH). Only the major states are shown: *core-contact*, *core-no-contact*, *portal-only*, and *dry-cavity*, as defined in the Molecular Dynamics section. For each state, the table reports the number of frames, population, mean total number of bridge waters, fraction of frames containing at least one bridge water, mean numbers of UU and UV hydrogen bonds, mean number of core waters, and mean number of core-contact waters. Low-population states were omitted for clarity. . S31

S32 Bridge-summary analysis for the Tyr@CB7 replicas initiated without a cavity water molecule (nHOH). Only the major states are shown: *core-contact*, *core-no-contact*, *portal-only*, and *dry-cavity*, as defined in the Molecular Dynamics section. For each state, the table reports the number of frames, population, mean total number of bridge waters, fraction of frames containing at least one bridge water, mean numbers of UU and UV hydrogen bonds, mean number of core waters, and mean number of *core-contact* waters. Low-population states were omitted for clarity. S32

S33 Per-frame water statistics for the Tyr@CB7 trajectories. Reported values were calculated over the full 300 ns trajectories (30 000 analysed frames per replica). The table summarizes the mean numbers of core and portal waters, the mean number of core-contact waters, and the fractions of frames containing core or portal waters. S32

S34 Summary of the full MD analysis for DOPA@CB7. The table compares the mean axial position of the ammonium nitrogen (N_α), mean axial position of the ligand center of geometry (COG), mean acute angle between the aromatic ring plane and the CB7 mid-plane, and the populations of the major water/positioning states identified in the trajectory analysis. S32

S35 State-resolved geometry for DOPA@CB7. Only the major MD-derived hydration states are shown: *core-contact*, *portal-only*, and *dry-cavity*, as defined in the Molecular Dynamics section. Reported values are mean ± standard deviation calculated over frames assigned to a given state. Low-population states were omitted for clarity. S32

S36 Bridge-summary analysis for DOPA@CB7. Only the major states are shown: *core-contact*, *portal-only*, and *dry-cavity*. For each state, the table reports the number of frames, population, mean total number of bridge waters, fraction of frames containing at least one bridge water, mean numbers of UU and UV hydrogen bonds, mean number of core waters, and mean number of core-contact waters. Low-population states were omitted for clarity. S32

S37 Summary of the full MD analysis for Trp@CB7. The table compares the mean axial position of the ammonium nitrogen (N_α), mean axial position of the ligand center of geometry (COG), mean acute angle between the aromatic ring plane and the CB7 mid-plane, and the populations of the major water/positioning states identified in the trajectory analysis. S33

S38 State-resolved geometry for Trp@CB7. Only the major MD-derived hydration states are shown: *core-contact*, *portal-only*, and *dry-cavity*, as defined in the Molecular Dynamics section. Reported values are mean ± standard deviation calculated over frames assigned to a given state. Low-population states were omitted for clarity, except for *core-contact*, which is included because it represents the direct water–ammonium contact state. S33

S39 Bridge-summary analysis for Trp@CB7. Only the major states are shown: *core-contact*, *portal-only*, and *dry-cavity*. For each state, the table reports the number of frames, population, mean total number of bridge waters, fraction of frames containing at least one bridge water, mean numbers of UU and UV hydrogen bonds, mean number of core waters, and mean number of core-contact waters. Low-population states were omitted for clarity, except for *core-contact*, which is included because it represents the direct water–ammonium contact state. S33
S40 Geometric descriptors for optimized CB7–amino acid complexes derived from crystal structures. One optimized geometry is shown for each guest/state. N_α and ligand COG positions were measured along the portal 1→portal 2 axis; the ring angle is given relative to the CB7 mid-plane. S34
S41 Counterpoise-corrected pairwise interaction energies for selected contacts in XRD-derived CB7–amino acid clusters. Energies are given in kJ mol^{-1}. The number of crystallographic water molecules included in each optimized cluster is indicated in the model name. Here, q_{AA} denotes the formal charge of the amino acid fragment. Wc denotes a cavity water molecule, whereas Wb denotes a bridge water molecule. Dashes indicate that the corresponding water molecule was not present in the selected cluster or that the pair interaction was not evaluated. S35
S42 Counterpoise-corrected pairwise interaction energies for selected contacts in representative MD-derived hydration states of Phe@CB7 and Tyr@CB7. Energies are given in kJ mol^{-1}. W1 denotes the state-defining water molecule selected for the local interaction analysis: the ammonium-contact cavity water in the *core-contact* state, the non-contact cavity water in the *core-no-contact* state, or the selected portal water in the *portal-only* state. Wb denotes an additional bridge water molecule involved in the local hydration network. $\Sigma E_{\mathrm{local}}$ is the sum of the listed pairwise interaction energies for a given cluster. Dashes indicate that the corresponding water molecule was not present in the selected cluster or that the pair interaction was not evaluated. S36
S43 State-resolved force-field interaction-energy components for Phe@CB7 and Tyr@CB7. Energies are reported as mean values in kJ mol^{-1}. For the *core-contact* states, W denotes the ammonium-contact cavity water and the averages were calculated over all frames assigned to this state. For the *portal-only* states, W denotes the selected portal water in representative frames ($n = 50$); for Tyr this is the OH-associated portal water, whereas for Phe it is the nearest ligand-associated portal water. $n_{\mathrm{W,unique}}$ denotes the number of unique water molecules contributing to the analysis. $E_{\mathrm{local}} = E_{\mathrm{CB7-AA}} + E_{\mathrm{AA-W}} + E_{\mathrm{CB7-W}}$. S36

1 Materials and buffer preparation for ITC

Cucurbit[7]uril (CB7) was purchased from BLD Pharm. L-Phenylalanine and L-tyrosine were purchased from Alfa Aesar Chemicals. L-Tryptophan, L-methionine, and L-DOPA were purchased from Merck/Sigma-Aldrich. L-Leucine was purchased from Thermo Scientific. All solvents used for crystallization were purchased from Chempur (Poland). All chemicals were of reagent or analytical grade and were used without further purification. Distilled water was used for the preparation of all solutions.

Glacial acetic acid (99.5–99.9%) and hydrochloric acid (35–38%) were purchased from POCH S.A. (Poland). Anhydrous sodium dihydrogen phosphate, anhydrous disodium hydrogen phosphate, sodium chloride, sodium hydroxide, and tris(hydroxymethyl)aminomethane (TRIS) were obtained from Chempur (Poland). Anhydrous sodium acetate and glycine were supplied by Merck (Germany). Piperazine-N,N′-bis(2-ethanesulfonic acid) (PIPES) was purchased from Chemat (Poland).

All ITC solutions were prepared in the corresponding buffer at the indicated pH. The same buffer was used for the CB7 solution and the amino acid solution in each titration. The pH was measured using a calibrated pH meter and, where necessary, adjusted with dilute HCl or NaOH.

Acetate buffer at pH 4.7 (20 mM) was prepared by dissolving 0.015 g of anhydrous sodium acetate and 0.013 g of acetic acid in distilled water, followed by adjustment of the final volume to 20 mL.

PIPES buffer at pH 6.5 was prepared by dissolving 0.121 g of PIPES and 0.148 g of sodium chloride in distilled water, followed by adjustment of the final volume to 20 mL. PIPES buffer at pH 7.5 was prepared analogously using 0.121 g of PIPES and 0.118 g of sodium chloride in a final volume of 20 mL.

Phosphate buffer at pH 7.4 was prepared by dissolving 0.048 g of anhydrous sodium dihydrogen phosphate and 0.119 g of sodium chloride in distilled water, followed by adjustment of the final volume to 20 mL. Phosphate buffer at pH 7.0 was prepared analogously using 0.048 g of anhydrous sodium dihydrogen phosphate and 0.127 g of sodium chloride in a final volume of 20 mL.

TRIS buffer at pH 7.5 was prepared by dissolving 0.048 g of TRIS and 0.164 g of sodium chloride in distilled water, followed by adjustment of the final volume to 20 mL.

TRIS buffer at pH 8.5 was prepared analogously using 0.048 g of TRIS and 0.176 g of sodium chloride in a final volume of 20 mL.

2 Crystallization

Crystals of the complexes were obtained by crystallization from saturated solutions of CB7 with selected amino acids. All crystals were obtained by dissolving CB7 and the selected amino acid in appropriate aqueous H_2SO_4 solution at elevated temperature. The obtained solutions were then slowly cooled and evaporated to form crystals. The crystal growth period ranged from a few days to about two weeks. The crystallization conditions are summarised in Table S1.

Table S1: Summary of crystallization conditions. CB7 0.007 mmol, 1 equiv.

Entry	Host	Ligand	Ligand/Host Ratio	Solvent	pH	Temperature (°C)
1	CB7	Phe	8:1	H_2O, H_2SO_4	4	70
2	CB7	Tyr	8:1	H_2O, H_2SO_4	2	40
3	CB7	DOPA	8:1	H_2O, H_2SO_4	4	80
4	CB7	Trp	8:1	H_2O, H_2SO_4	4	40
5	CB7	Met	4:1	H_2O, H_2SO_4	2	60
6	CB7	Leu	4:1	H_2O, H_2SO_4	2	60

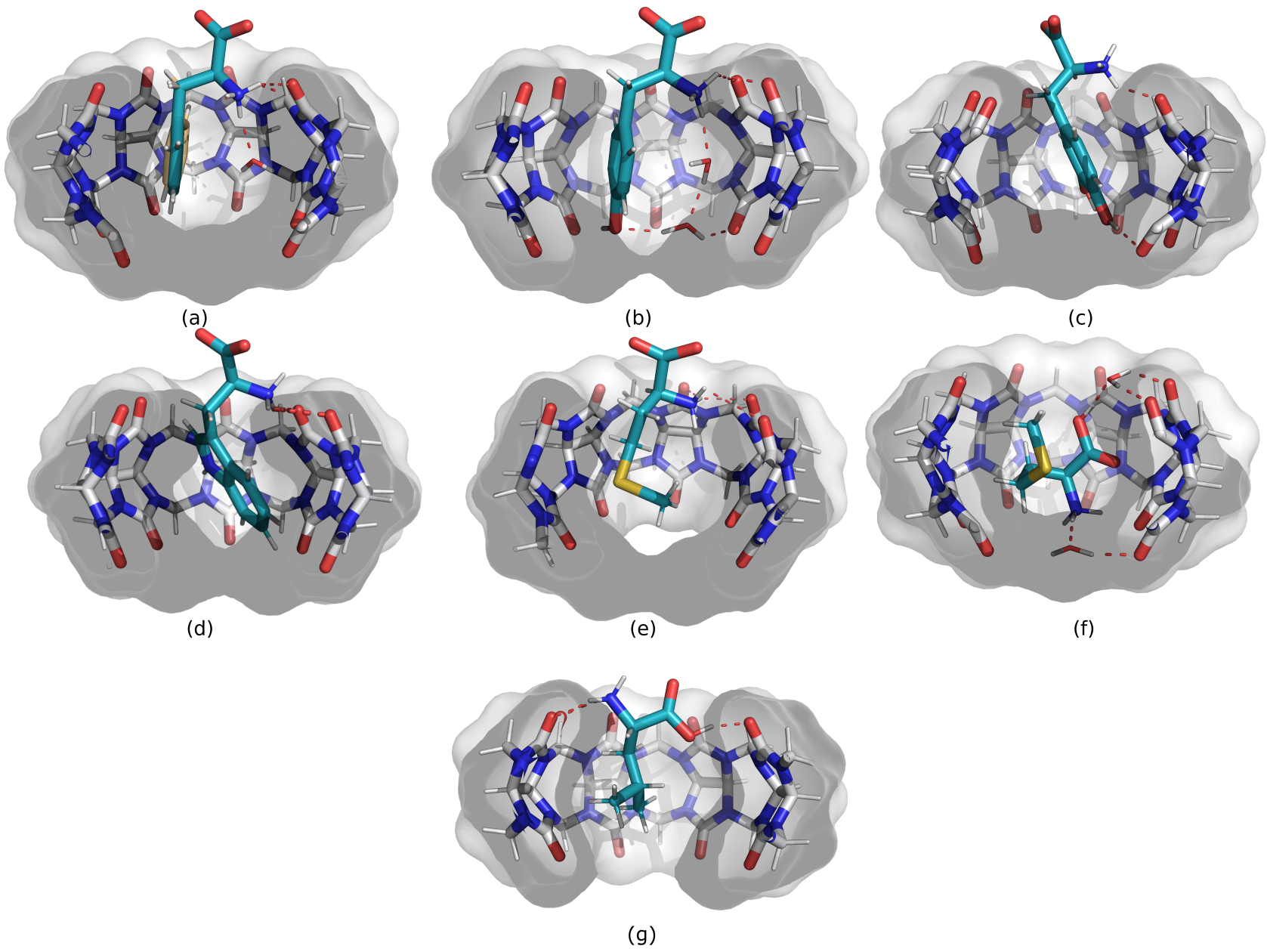

Figure S1: General overview of the binding pose of Phe (charge 0) (a), Tyr (charge 0) (b), DOPA (charge 0) (c), Trp (charge 0) (d), Met (charge 0) (e), Met (charge +1) (f), Leu (charge +1) (g) in the CB7 cavity. The CB7 molecule is depicted using a stick representation, and the van der Waals surfaces are shown. The CB7 molecule is clipped to show the amino acid position. Hydrogen bonds are represented as red dashed lines.

3 X-ray diffraction

X-ray diffraction (XRD) data were collected using colourless, transparent, and well-formed crystals on an Agilent SuperNova diffractometer, which was controlled by the CrysAlis PRO software and equipped with a Cu Kα micro-focus X-ray source (λ = 1.54 Å, 50.0 kV, and 0.8 mA) and either an Atlas or a HyPix detector. The experiments were carried out at 100.0(2) K controlled using an Oxford Cryosystems cooling device. The crystals were placed on a MiTeGen mount with a droplet of immersion oil and immediately cooled. Each crystal was positioned 54.0 mm from the detector. Indexing and integration were performed using the CrysAlis PRO software. The structures were solved using SHELXT[S1] and refined using SHELXL.[S2] Refinement was based on F^2 for all reflections. Weighted R-factors (wR) and all goodness-of-fit values (S) were based on F^2, whereas conventional R-factors were based on amplitudes, with F set to zero for negative F^2. The atomic scattering factors were obtained from the International Tables for Crystallography.[S3] Data collection and processing statistics are summarised in Tables S2 and S3. Figures S2–S5 show the asymmetric units with ADPs for non-hydrogen atoms.

Table S2: Experimental details for structures **1**–**3**. Experiments were carried out at 100 K with Cu Kα radiation.

	**1** (Phe)	**2** (Tyr)	**3** (DOPA)
**Crystal data**			
Chemical formula	$C_{42}H_{42}N_{28}O_{14} \cdot 2\,C_9H_{11}NO_2 \cdot 20\,H_2O$	$3\,C_9H_{11}NO_3 \cdot 4\,C_{42}H_{42}N_{28}O_{14} \cdot 106\,H_2O$	$C_{42}H_{42}N_{28}O_{14} \cdot 2\,C_9H_{11}NO_4 \cdot 15\,H_2O$
M_r	1853.73	7105.38	1827.65
Crystal system, space group	Orthorhombic, $P2_12_12_1$	Monoclinic, $P2_1$	Orthorhombic, $P2_12_12_1$
a, b, c (Å)	15.11797(10), 17.08103(12), 31.3004(2)	25.1436(2), 13.6434(1), 45.2737(3)	14.95655(9), 17.13985(9), 30.38789(17)
α, β, γ (°)	90, 90, 90	90, 102.614(1), 90	90, 90, 90
V (Å^3)	8082.71(9)	15156.0(2)	7790.03(8)
Z	4	2	2
μ (mm^{-1})	1.10	1.18	1.12
Crystal size (mm)	0.23 × 0.17 × 0.10	0.56 × 0.43 × 0.13	0.25 × 0.19 × 0.04
**Data collection**			
Diffractometer	SuperNova, Dual, Atlas	SuperNova, Dual, HyPix	SuperNova, Dual, Atlas
Absorption correction	Multi-scan	Multi-scan	Multi-scan
$T_{\min}, T_{\max}$	0.459, 1.000	0.564, 1.000	0.748, 1.000
No. of measured, independent and observed [$I > 2\sigma(I)$] reflections	143844, 16332, 16175	219840, 59016, 42299	206112, 16420, 15946
R_{int}	0.034	0.038	0.041
$(\sin\theta/\lambda)_{\max}$ (Å^{-1})	0.624	0.620	0.632
Resolution, $d_{\min}$ (Å)	0.80	0.81	0.79
**Refinement**			
$R[F^2 > 2\sigma(F^2)]$, $wR(F^2)$, S	0.038, 0.106, 1.11	0.057, 0.184, 1.05	0.052, 0.150, 1.04
No. of reflections	16332	59016	16420
No. of parameters	1252	3409	1195
No. of restraints	178	18	3
$\Delta\rho_{\max}, \Delta\rho_{\min}$ (e Å^{-3})	0.83, -0.43	0.60, -0.33	0.90, -0.62
Absolute structure parameter	0.00(2)	0.50(5)	0.02(2)
CCDC deposition number	2571857	2571856	2571854

Table S3: Experimental details for structures **4–6**. Experiments were carried out at 100 K with Cu Kα radiation.

	4 (Trp)	5 (Met)	6 (Leu)
**Crystal data**			
Chemical formula	$C_{42}H_{42}N_{28}O_{14} \cdot 2\,C_{11}H_{12}N_2O_2 \cdot 110\,H_2O$	$2\,C_5H_{12}NO_2S \cdot 4\,C_{42}H_{42}N_{28}O_{14} \cdot C_5H_{11}NO_2S \cdot 50\,H_2O$	$Cl \cdot 38\,H_2O \cdot 2\,C_{42}H_{42}N_{28}O_{14} \cdot 2\,C_6H_{14}NO_2$
M_r	1929.66	5153.80	3310.48
Crystal system, space group	Orthorhombic, $P2_12_12_1$	Monoclinic, $P2_1$	Orthorhombic, $P2_12_12_1$
a, b, c (Å)	15.07289(12), 17.45424(15), 32.7824(3)	21.8020(3), 14.3773(2), 39.5435(4)	15.29146(8), 29.50561(18), 32.00065(14)
α, β, γ (°)	90, 90, 90	90, 92.185(1), 90	90, 90, 90
V (Å^3)	8624.58(13)	12386.1(3)	14438.18(13)
Z	4	2	4
μ (mm^{-1})	1.08	1.15	1.28
Crystal size (mm)	0.21 × 0.14 × 0.13	0.17 × 0.16 × 0.14	0.33 × 0.22 × 0.12
**Data collection**			
Diffractometer	SuperNova, Dual, Atlas	SuperNova, Dual, HyPix	SuperNova, Dual, Atlas
Absorption correction	Multi-scan	Multi-scan	Multi-scan
$T_{\min}, T_{\max}$	0.667, 1.000	0.662, 1.000	0.584, 1.000
No. of measured, independent and observed $[I > 2\sigma(I)]$ reflections	79723, 16382, 15774	225169, 47749, 38347	141650, 28940, 27750
R_{int}	0.031	0.049	0.027
$(\sin\theta/\lambda)_{\max}$ (Å^{-1})	0.610	0.610	0.624
Resolution, $d_{\min}$ (Å)	0.82	0.82	0.80
**Refinement**			
$R[F^2 > 2\sigma(F^2)]$, $wR(F^2)$, S	0.061, 0.181, 1.03	0.097, 0.301, 1.22	0.047, 0.131, 1.03
No. of reflections	16382	47749	28940
No. of parameters	1163	3325	1948
No. of restraints	0	14	4
$\Delta\rho_{\max}, \Delta\rho_{\min}$ (e Å^{-3})	0.76, -0.43	0.86, -0.83	1.23, -0.78
Absolute structure parameter	0.14(3)	0.222(10)	0.070(6)
CCDC deposition number	2571852	2571855	2571853

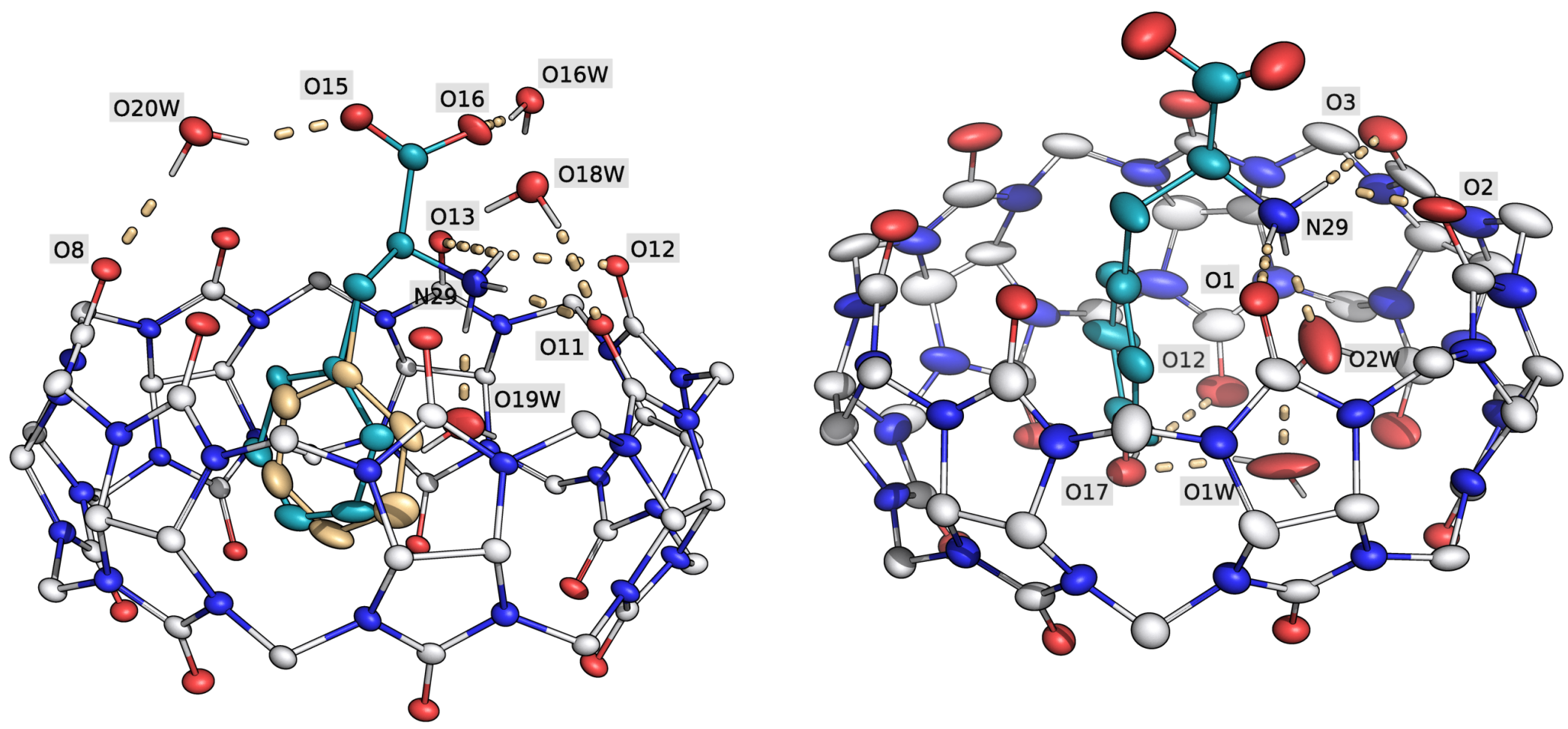

Figure S2: Phe (a) and Tyr (b) within the CB7 cavity, highlighting water molecules from the crystal structure **1** and **2**, respectively. Heavy atoms are displayed with anisotropic displacement parameters (ADPs), while only the positions of polar hydrogen atoms are shown. Labels indicate the positions of hydrogen bond donors and acceptors.

Single-crystal X-ray diffraction revealed inclusion complexes of CB7 with Phe (1), Tyr (2), DOPA (3), Trp (4), Met (5), and Leu (6). Because enantiomerically pure L-amino acids were used, all complexes crystallized in Sohncke space groups. The asymmetric units differ among the structures: Phe, DOPA, and Trp contain one crystallographically independent complex, whereas Tyr and Met contain four independent CB7 molecules, including one empty host, and Leu contains two independent CB7–Leu complexes.

The crystal structures of the selected CB7–amino acid complexes reveal both conserved and variable features that govern the binding geometry (Figures S1–S5). In all cases, the amino acid is bound with the α-ammonium group directed toward one CB7 portal, where it engages in ion–dipole and hydrogen-bonding interactions with the carbonyl rim. At the same time, the position of the side chain, the protonation state of the guest, and the hydration pattern within and around the cavity vary from one amino acid to another.

Crystallization was most successful from acidic aqueous solutions, typically in the pH range 2–4. Under these conditions, Phe, Tyr, DOPA, and Trp were observed as zwitterions, Leu as a cation, and Met in both zwitterionic and cationic forms within the same crystal structure.

To compare the positions of the ammonium and carboxyl/carboxylate groups across the crystal structures, a common geometric reference frame was defined based on the CB7 host (Figure S6). Cen1 is the centroid of the seven portal oxygen atoms at the portal interacting with the ammonium group, and Cen1′ is the corresponding centroid of the opposite portal. Cen2 is defined as the centroid of the fourteen equatorial carbon atoms of CB7. Plane 1 and Plane 1′ were defined as the average planes through the oxygen atoms of the two portals. The portal1→portal2 vector defines the axial coordinate used to project the positions of N_α, the carboxyl/carboxylate carbon atom, and the ligand center of geometry. In this convention, negative axial positions indicate displacement outside the CB7 cavity, whereas positive values indicate displacement toward the CB7 interior. For aromatic guests, the acute ring-plane angle was defined as the acute angle between the aromatic ring plane and the CB7 mid-plane.

Although the amino acids differ substantially in size and side-chain character, the position of the ammonium group is relatively conserved. In all complexes, the ammonium group is tilted toward the portal rim, generating one or two direct hydrogen bonds with portal carbonyl oxygen atoms (Table S4). The tilt is described by the $\angle$Cen1–Cen2–N_α angle and falls in a relatively narrow range. In well-resolved structures, the NH_3^+ group typically participates in four hydrogen

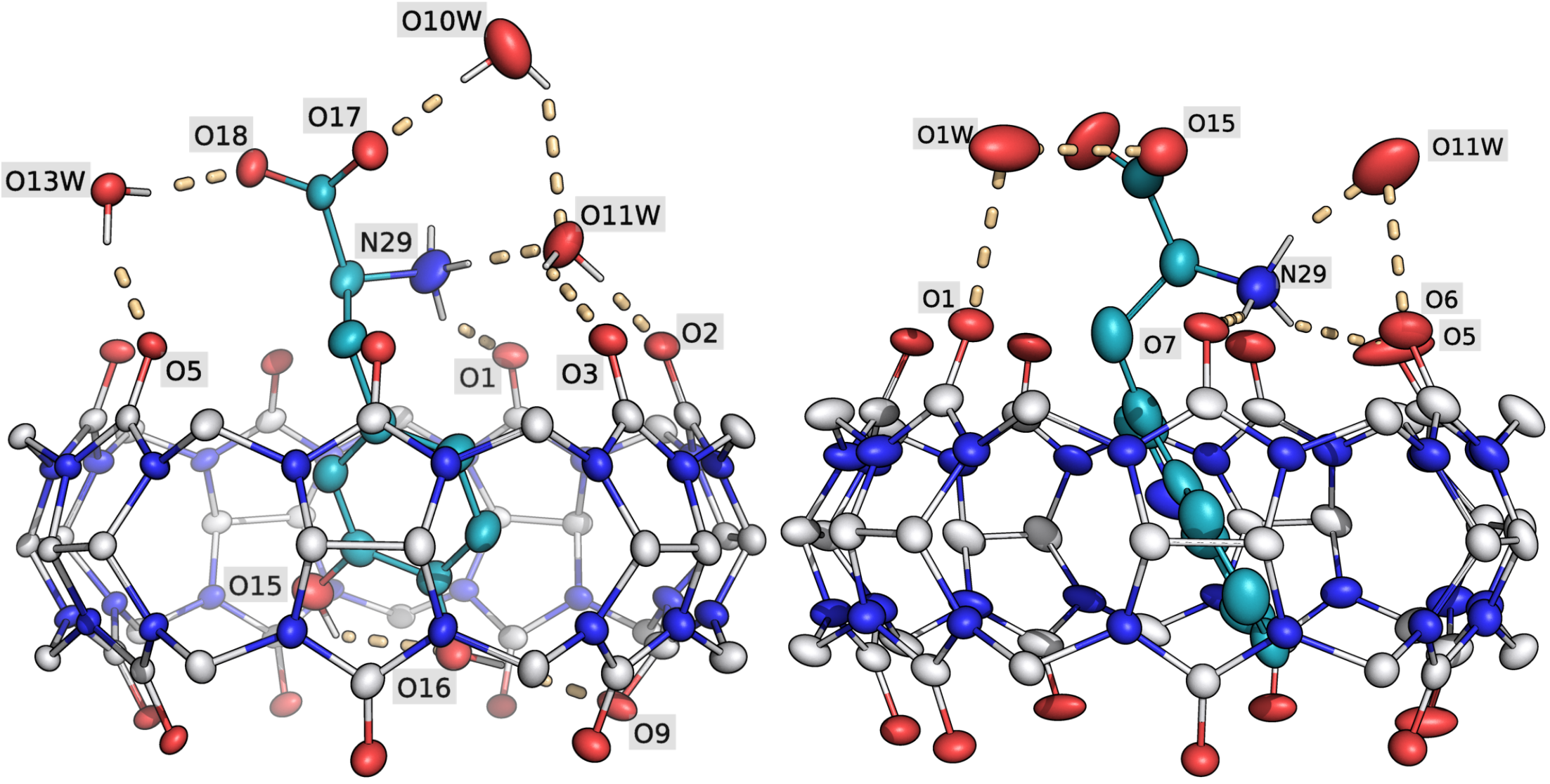

Figure S3: DOPA (a) and Trp (b) within the CB7 cavity, highlighting water molecules from the crystal structure **3** and **4**, respectively. Heavy atoms are displayed with anisotropic displacement parameters (ADPs), while only the positions of polar hydrogen atoms are shown. Labels indicate the positions of hydrogen bond donors and acceptors.

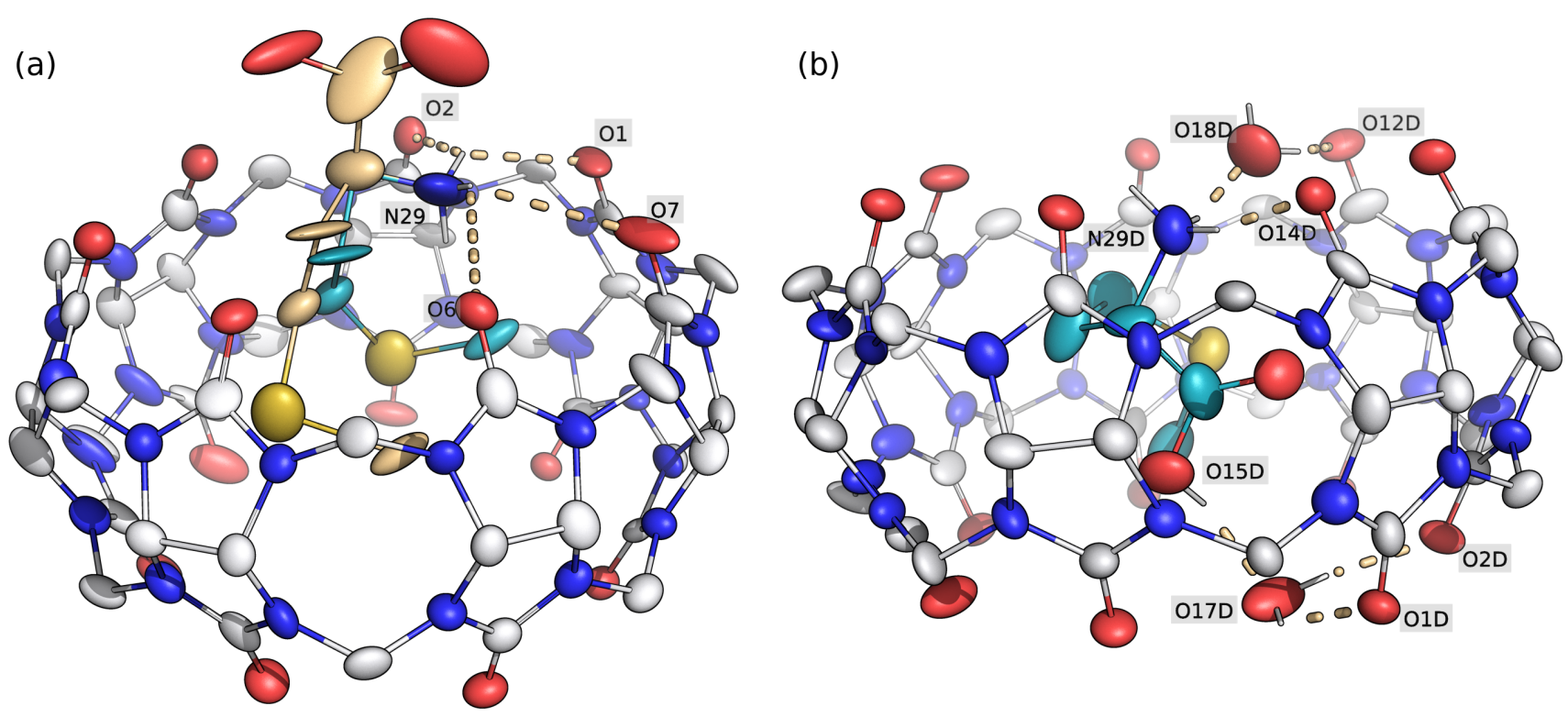

Figure S4: Met in zwitterionic form (a) and cationic form (b) within the CB7 cavity, highlighting water molecules from the crystal structure **5**. Heavy atoms are displayed with ADPs, while only the positions of polar hydrogen atoms are shown. Labels indicate the positions of hydrogen bond donors and acceptors.

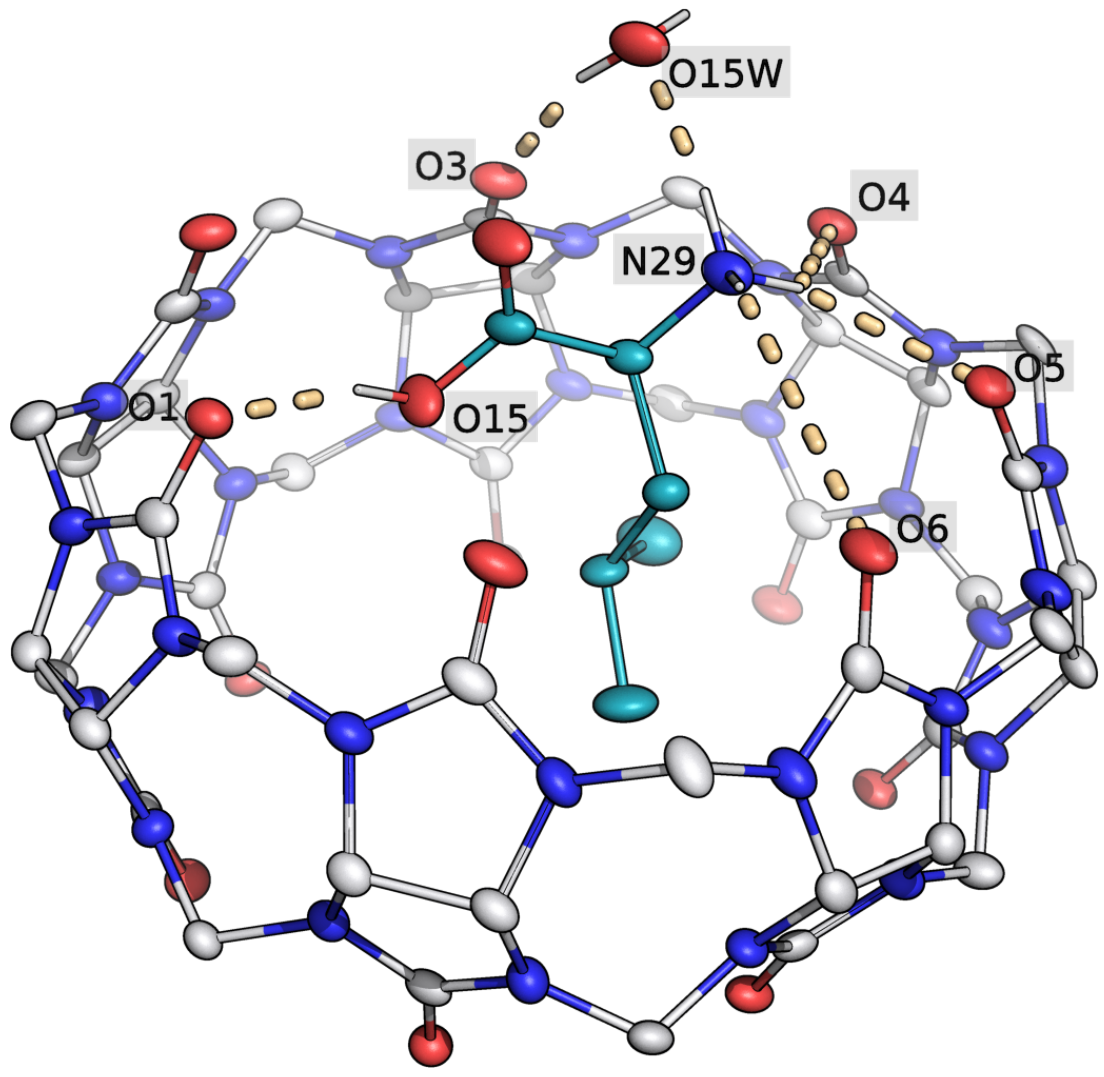

Figure S5: Leu in cationic form within the CB7 cavity, highlighting water molecules from the crystal structure **6**. Heavy atoms are displayed with anisotropic displacement parameters (ADPs), while only the positions of polar hydrogen atoms are shown. Labels indicate the positions of hydrogen bond donors and acceptors.

bonds in total, including interactions with water molecules. However, usually only two of these display nearly optimal geometry, with D–H. . . A angles close to or above 150°. At least one hydrogen bond is formed to a CB7 portal oxygen atom, although some of these contacts are relatively long. A complete list of hydrogen bonds is provided in Tables S6–S11.

In the X-ray structures, the ammonium group is anchored at the CB7 portal through ion–dipole and hydrogen-bonding interactions with the carbonyl rim. The signed distance between N_α and Plane 1 therefore provides a useful descriptor of the ammonium-group position relative to the portal plane. This distance ranges from -1.334 Å for DOPA to 0.687 Å for cationic Met (Table S5). Positive values indicate that the nitrogen atom is located below the portal plane, deeper toward the equatorial region of CB7, whereas negative values indicate a position above the portal plane, farther from the CB7 interior. Thus, this parameter distinguishes shallower and more deeply inserted ammonium binding modes in the crystal structures.

For amino acids observed in the zwitterionic form, the carboxylate group is consistently directed toward the exterior of CB7, which minimises unfavourable interactions with the electronegative portal region. The orientation of the C_α atom relative to the host is also similar across the series, indicating that the anchoring of the ammonium group largely determines the overall placement of the amino acid backbone. The main structural differences are therefore associated with the side-chain orientation and local hydration pattern.

The aromatic guests show distinct side-chain arrangements in the X-ray structures. In the Phe and Tyr complexes, where cavity water molecules are present, the aromatic rings are oriented nearly perpendicular to the CB7 portal plane. This geometry is observed consistently for the symmetry-independent Tyr complexes. In contrast, Trp and DOPA adopt more tilted orientations, with the aromatic ring plane forming an angle of about 60° with the portal plane. These observations indicate that cavity hydration is associated with the detailed positioning of the aromatic side chain inside CB7.

Water molecules play an important role in several of the crystal structures. In the Phe complex, one cavity water molecule is located next to the phenyl ring and accepts a hydrogen bond from the ammonium group, with an N29. . . O19W distance of 2.858(4) Å. In the Tyr complex, two cavity water molecules are observed; one accepts a hydrogen bond from the ammonium group at 2.762(8) Å, while the corresponding contact in another independent complex is slightly longer, 3.02(1) Å. In addition, one of the Tyr water molecules hydrogen-bonds to the second

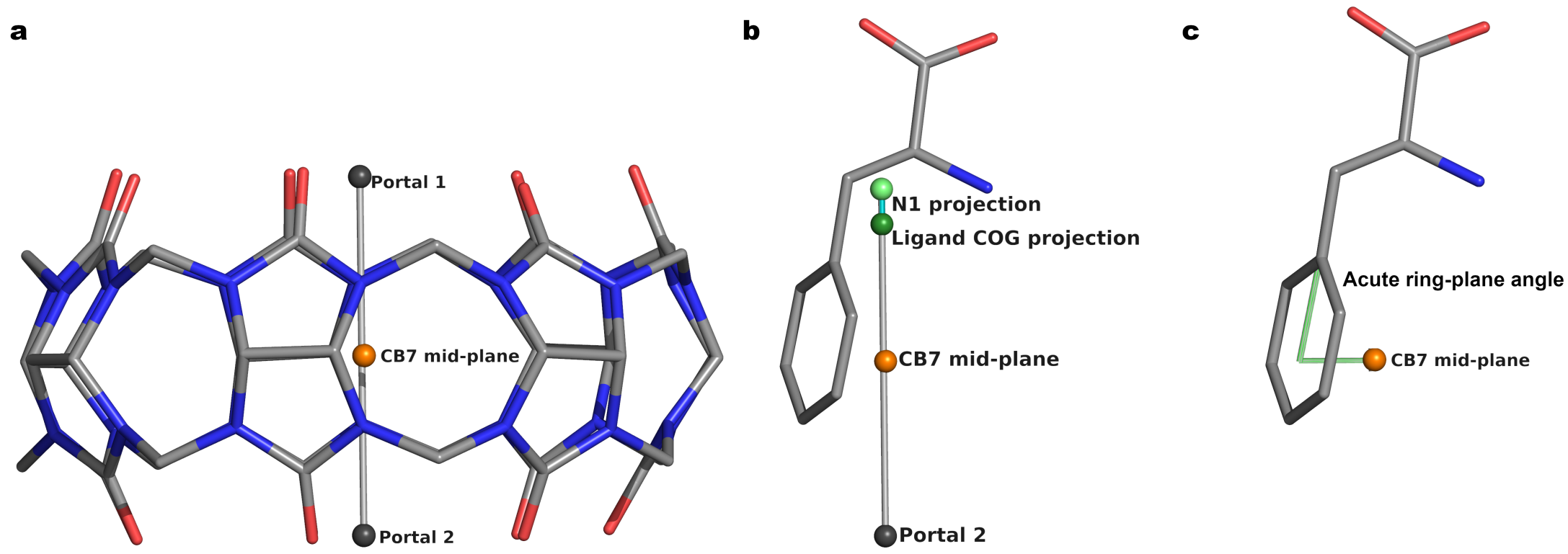

Figure S6: Definition of the geometric descriptors used in the analysis of CB7–amino acid complexes. (a) The CB7 reference frame is defined by the centroids of portal 1, portal 2, and the CB7 mid-plane; the portal1→portal2 vector defines the axial coordinate. (b) The projected positions of N_α and the ligand center of geometry (COG) are measured along this axis from portal 1. (c) For aromatic guests, the acute ring-plane angle is defined as the acute angle between the aromatic ring plane and the CB7 mid-plane.

water molecule. In contrast, no cavity water is observed for DOPA, Trp, Met, or Leu.

Besides cavity water, external bridging water molecules are also found in several crystal structures. These waters extend the hydrogen-bonding network outside the CB7 cavity by mediating contacts between the amino acid functional groups and the CB7 portal carbonyls. For Phe, DOPA, and Trp, the bridging waters are associated mainly with the carboxylate/portal region. In Met, this motif is observed for the cationic binding modes, where bridging waters connect either the carboxyl/carboxylate region or the ammonium group with the CB7 portal region, depending on the symmetry-independent complex. In Leu, which is observed in the cationic form, the bridging waters are associated mainly with the ammonium/portal region rather than with the carboxyl group.

Overall, the crystal structures show a conserved anchoring of the ammonium group at one CB7 portal, while the side-chain orientation, hydration pattern, and protonation state remain guest-dependent.

Table S4: Geometric parameters describing the orientation of the amino acid backbone relative to the CB7 host in crystal structures **1**–**6**. The geometry is characterized by the tilt of the ammonium group, defined by the angle Portal 1–CB7 midpoint–N_α, the tilt of the carboxyl/carboxylate group, defined by the angle Portal 1–CB7 midpoint–C, and the tilt of the α-carbon, defined by the angle Portal 1–CB7 midpoint–C_α. Atom labels are retained to distinguish symmetry-independent complexes in the asymmetric unit.

Crystal structure	AA	Charge	Portal 1–CB7 midpoint–N_α Atom	(°)	Portal 1–CB7 midpoint–C Atom	(°)	Portal 1–CB7 midpoint–C_α Atom	(°)
1	Phe	0	N29	31.67	C44	10.65	C45	13.76
2	Tyr	0	N29	35.09	C44	13.35	C45	12.61
		0	N29C	28.33	C44C	18.03	C45C	18.73
		0	N29D	33.01	C44D	14.92	C45D	13.00
3	DOPA	0	N29	18.97	C44	12.83	C45	13.61
4	Trp	0	N30	26.08	C44	5.86	C45	15.07
5	Met	0	N29	26.69	C44	15.75	C45A/C45B	16.91/13.14
		1	N29C	33.18	C44C	46.44	C45C	35.59
		1	N29D	28.27	C44D	57.32	C45D	67.10
6	Leu	1	N29	23.07	C44	16.32	C45	16.32
		1	N29A	23.47	C44A	18.92	C45A	32.45

Table S5: Geometric parameters for each ligand position in crystal structures **1**–**6**, describing the binding geometry relative to portal 1 (P1). The first parameter is the signed perpendicular distance between the nitrogen atom of the amine group and the average plane of portal 1, denoted as $d(\mathrm{P1} \ldots \mathrm{N})$. Negative values indicate that the atom is located above the portal plane, that is, farther from the CB7 midpoint, whereas positive values indicate that it lies below the portal plane, toward the CB7 interior. The second parameter is the analogous signed perpendicular distance for the carboxyl carbon atom, $d(\mathrm{P1} \ldots \mathrm{C})$. The third parameter is the angle between the P1 plane and the average plane of the aromatic ring. For amino acids without aromatic side chains, this parameter is not defined. All geometric parameters in this table were measured in Mercury from the crystallographic model.

Crystal structure	AA	Charge	$d(\mathrm{P1} \ldots \mathrm{N})$ (Å)		$d(\mathrm{P1} \ldots \mathrm{C})$ (Å)		$\angle(\mathrm{P1} \ldots \mathrm{ringA})$ (°)
1	Phe	0	N29	-0.005	C44	0.624	85.16/82.59
2	Tyr	0	N29	0.248	C44	0.466	85.62
2		0	N29C	0.017	C44C	0.320	89.77
2		0	N29D	0.398	C44D	0.338	89.64
3	DOPA	0	N29	-1.334	C44	1.183	65.57
4	Trp	0	N29	-0.755	C44	1.176	61.85
5	Met	0	N29	-0.055	C44	0.475	n/a
5		1	N29C	0.520	C44C	-1.646	n/a
5		1	N29D	0.687	C44D	2.026	n/a
6	Leu	1	N29	-0.820	C44	-0.321	n/a
6		1	N29A	-0.743	C44A	-0.426	n/a

Table S6: Selected Hydrogen-Bond Parameters for the Crystal Structure of the Complex Between CB7 and Phe (**1**)

D	H	A	H···A (Å)	D···A (Å)	D–H···A (°)
N30	H30C	O18W[1]	1.98	2.782(3)	146.7
N30	H30D	O3[2]	1.98	2.857(3)	162.0
N30	H30E	O12[1]	1.94	2.837(3)	168.0
O18W	H18C	O11	2.32	2.798(3)	116.2
O16W	H16A	O16	1.99	2.819(3)	163.5
O20W	H20B	O8	2.04	2.883(3)	175.8
O20W	H20A	O15	1.95	2.743(4)	155.5
C14	H14	O6[3]	2.27	3.083(3)	137.8
C14	H14	O5[3]	2.4	2.904(4)	110.2
C9	H9A	O17[4]	2.53	3.282(4)	132.7
C7	H7	O20W[5]	2.47	3.270(4)	136.5
C3W	H3WA	O10W[6]	2.65	3.560(4)	152.7
C27	H27	O18	2.63	3.381(3)	131.9
N29	H29C	O12	2.21	2.807(3)	122.9
N29	H29C	O13	2.44	2.958(4)	116.1
N29	H29D	O19W	1.96	2.858(4)	169.0
N29	H29E	O11	2.18	3.043(4)	158.5
C24	H24A	O2[3]	2.45	3.112(3)	124.1
C18	H18B	O4[3]	2.33	3.162(4)	141.3
C10	H10B	O14W[3]	2.48	3.375(4)	149.9
C23	H23A	O2W	2.51	3.190(4)	125.6
C23	H23B	O4W	2.55	3.348(4)	137.3
C23	H23B	O6W	2.64	3.560(4)	154.8
C36	H36B	O3W[6]	2.59	3.288(4)	127.9
C21	H21	O2[3]	2.52	3.248(3)	129.8
C39	H39	O3W[6]	2.35	3.102(4)	131.2
C33	H33	O12W[1]	2.5	3.112(4)	119.4
C17	H17A	O7[3]	2.61	3.348(4)	131.6
C12	H12A	O15[5]	2.47	3.435(4)	164.7
C45	H45A	O10	2.54	3.420(4)	148.5
C2	H2	O16[5]	2.65	3.630(4)	165.5
C26	H26	O6W	2.5	3.404(4)	149.9
C29	H29B	O18	2.34	3.253(4)	152.3
C3	H3	O10W[6]	2.65	3.467(4)	138.7
C15	H15	O14W[3]	2.34	3.223(4)	146.9

[1] -1/2+X,3/2-Y,1-Z;
[2] +X,1+Y,+Z;
[3] 1/2+X,1/2-Y,1-Z;
[4] +X,-1+Y,+Z;
[5] 1-X,-1/2+Y,3/2-Z;
[6] 1/2-X,1-Y,1/2+Z

Table S7: Selected Hydrogen-Bond Parameters for the Crystal Structure of the Complex Between CB7 and Tyr (**2**)

D	H	A	H···A (Å)	D···A (Å)	D–H···A (°)
O17	H17	O13	2.4	2.882(6)	117.1
O17C	H17I	O2C	2.15	2.990(10)	176.6
N29	H29L	O3	2.47	2.989(7)	116.7
N29	H29L	O2	2.04	2.729(7)	131
N29	H29M	O2W	1.89	2.762(8)	159.5
N29	H29N	O1	2.18	3.080(7)	169.7
N29D	H29O	O4D	2.05	2.655(7)	122.5
N29D	H29O	O15D	2.22	2.722(12)	113.9
N29D	H29P	O3D	2.65	3.269(7)	126.3
N29C	H29I	O3W	2.31	3.025(14)	134.8
N29C	H29I	O3X	1.95	2.816(10)	157.2
N29C	H29J	O10C	2.52	3.156(9)	127.2
N29C	H29K	O11C	1.91	2.716(7)	146.9

Table S8: Selected Hydrogen-Bond Parameters for the Crystal Structure of the Complex Between CB7 and DOPA (**3**)

D	H	A	H···A (Å)	D···A (Å)	D–H···A (°)
O19	H19	O22[1]	1.78	2.593(4)	163.5
N30	H30C	O8[2]	1.91	2.812(4)	168.8
N30	H30D	O6[3]	1.87	2.761(4)	167.7
N30	H30E	O18[3]	2.08	2.815(4)	137
O20	H20A	O3W[4]	1.82	2.660(5)	178.4
O16	H16	O9	2.02	2.851(5)	168.1
O15	H15A	O16	2.19	2.662(5)	115.9
N29	H29C	O1	1.99	2.766(5)	142
N29	H29D	O11W	1.86	2.751(7)	166.8
N29	H29E	O9W	2.07	2.975(8)	173.3

[1] 1/2+X,1/2-Y,-Z;-Z;
[2] -1/2+X,-1/2-Y,
[3] -1+X,+Y,+Z;
[4] 1-X,1/2+Y,1/2-Z

Table S9: Selected Hydrogen-Bond Parameters for the Crystal Structure of the Complex Between CB7 and Trp (**4**)

D	H	A	H···A (Å)	D···A (Å)	D–H···A (°)
N30	H30	N13	2.47	3.127(6)	131.9
N29	H29C	O7	2.63	3.475(7)	155.5
N29	H29D	O11W	1.82	2.69(2)	158.8
N29	H29D	O10W	2.00	2.792(8)	144.1
N29	H29E	O5	2.27	3.101(8)	151.9
N32	H32A	O4W[1]	1.83	2.731(6)	170
N32	H32B	O15	1.93	2.780(7)	155.8
N32	H32C	O7	1.88	2.749(5)	159.7

[1] 1+X,+Y,+Z

Table S10: Selected Hydrogen-Bond Parameters for the Crystal Structure of the Complex Between CB7 and Met (**5**)

D	H	A	H···A (Å)	D···A (Å)	D–H···A (°)
N29C	H29L	O2C	2.46	3.241(16)	143.8
N29C	H29M	O1C	2.01	2.851(13)	153.2
N29D	H29O	O14D	1.88	2.766(12)	164.2
N29D	H29Q	O18D	1.89	2.753(15)	156.7
N29	H29J	O2	2.28	2.89(3)	123.9
N29	H29J	O1	2.32	2.94(2)	124.4
N29	H29K	S1B	2.70	3.53(3)	151.3

Table S11: Selected Hydrogen-Bond Parameters for the Crystal Structure of the Complex Between CB7 and Leu (**6**)

D	H	A	H···A (Å)	D···A (Å)	D–H···A (°)
O15	H15B	O1	1.88	2.695(4)	162.2
N29	H29E	O4	2.14	2.754(4)	124
N29	H29E	O5	2.35	3.098(4)	139.7
N29	H29F	O20W	1.93	2.820(5)	167.3
N29	H29G	O15W	1.94	2.830(5)	164
O15A	H15C	O1A	2.46	3.156(4)	141.3
O15A	H15C	O7A	2.19	2.789(4)	127.9
N29A	H29H	O6W	1.98	2.870(4)	166.6
N29A	H29I	O11W	1.97	2.864(4)	167.9
N29A	H29J	O4A	2.04	2.923(4)	163.8

4 Protonation linkage of binding affinities

The apparent affinity of amino acids toward CB7 depends on the protonation state of the ligand. In the present model, three acid–base forms of the amino acid are considered: the cationic form, L^+, the zwitterionic form, L^0, and the anionic form, L^-. Each form is assigned its own microscopic association constant with CB7, denoted as K_b^{+1}, K_b^0, and K_b^{-1}, respectively. The zwitterionic form, L^0, was selected as the reference state.

The protonation equilibria considered in the model are:

$$L^0 + H^+ \rightleftharpoons L^+ \tag{1}$$

for protonation of the carboxylate group, and

$$L^- + H^+ \rightleftharpoons L^0 \tag{2}$$

for protonation of the amino group. The corresponding protonation constants are denoted as $K_{p,\mathrm{COOH}}$ and $K_{p,\mathrm{NH_3}}$, respectively. Superscripts f and b refer to the free and CB7-bound ligand.

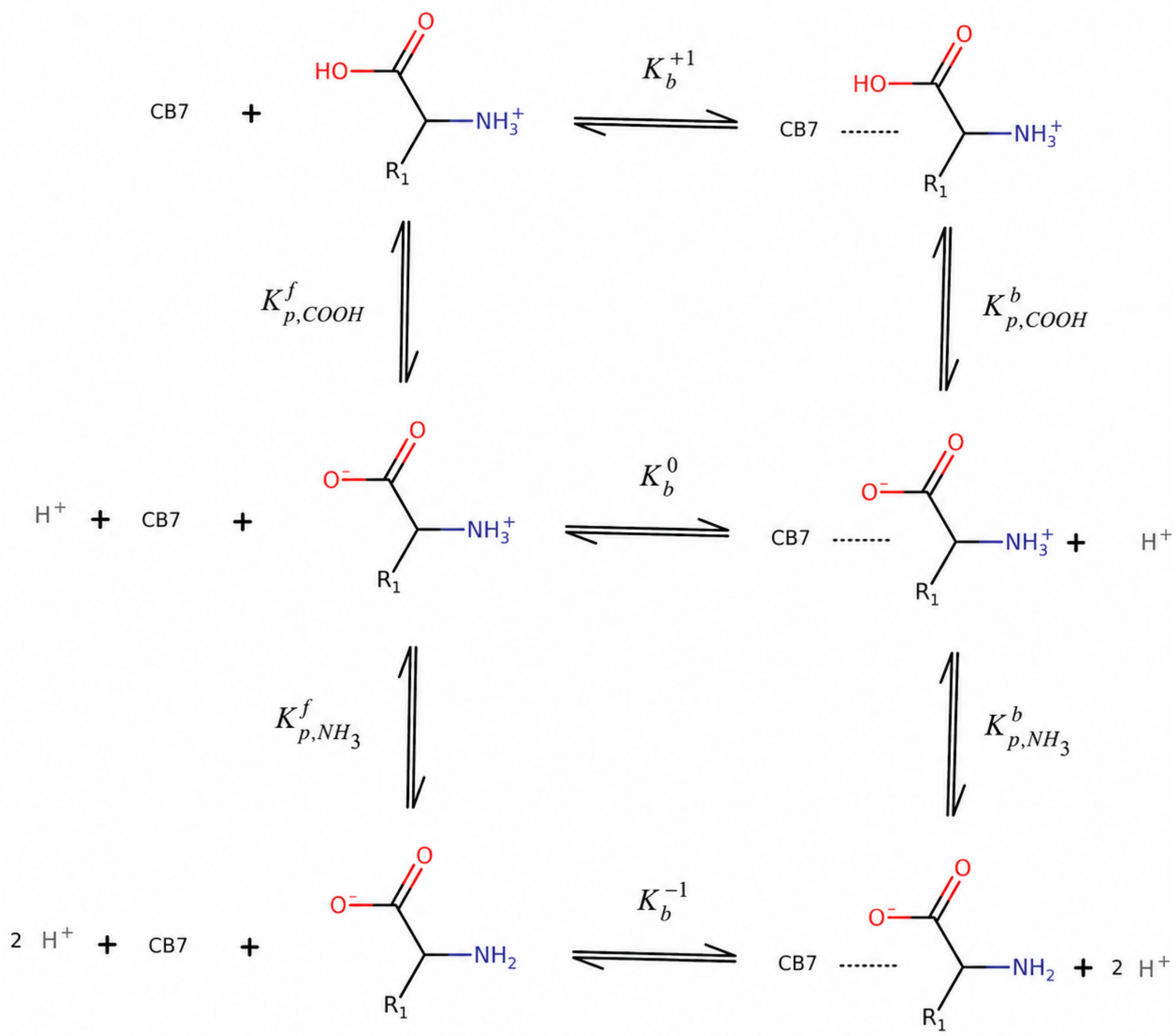

Figure S7: Thermodynamic linkage scheme describing CB7 binding coupled to the protonation equilibria of the amino acid.

Because binding and protonation form closed thermodynamic cycles, the microscopic binding constants are related to the protonation constants of the free and bound ligand. For the cationic/zwitterionic equilibrium:

$$K_b^{+1} K_{p,\mathrm{COOH}}^f = K_b^0 K_{p,\mathrm{COOH}}^b \tag{3}$$

and for the zwitterionic/anionic equilibrium:

$$K_b^0 K_{p,\mathrm{NH_3}}^f = K_b^{-1} K_{p,\mathrm{NH_3}}^b \tag{4}$$

The observed association constant, K_{obs}, measured at a given pH, is therefore a population-weighted quantity that depends on the distribution of protonation states in both the free and bound forms. Using K_b^0 as the reference binding constant and $a_{H^+} = 10^{-\mathrm{pH}}$, the apparent binding constant can be written as:

$$K_{\mathrm{obs}} = K_b^0 \cdot \frac{1 + 10^{\left(\mathrm{p}K_{\mathrm{a,COOH}}^b - \mathrm{pH}\right)} + 10^{\left(\mathrm{pH} - \mathrm{p}K_{\mathrm{a,NH_3}}^b\right)}}{1 + 10^{\left(\mathrm{p}K_{\mathrm{a,COOH}}^f - \mathrm{pH}\right)} + 10^{\left(\mathrm{pH} - \mathrm{p}K_{\mathrm{a,NH_3}}^f\right)}} \tag{5}$$

Here, $\mathrm{p}K_{\mathrm{a,COOH}}^f$ and $\mathrm{p}K_{\mathrm{a,NH_3}}^f$ describe the acid–base equilibria of the free amino acid, whereas $\mathrm{p}K_{\mathrm{a,COOH}}^b$ and $\mathrm{p}K_{\mathrm{a,NH_3}}^b$ describe the corresponding equilibria in the CB7-bound state. Thus, deviations of K_{obs} from K_b^0 reflect changes in the protonation-state populations induced by complex formation.

The net number of protons taken up or released upon binding, n_H, can be calculated from the difference between the average protonation state of the bound and free ligand:

$$n_H = \nu_b - \nu_f \tag{6}$$

where ν_b and ν_f are the average numbers of protons associated with the bound and free ligand relative to the zwitterionic reference state. These quantities are given by:

$$\nu_b = \frac{10^{\left(\mathrm{p}K_{\mathrm{a,COOH}}^b - \mathrm{pH}\right)} - 10^{\left(\mathrm{pH} - \mathrm{p}K_{\mathrm{a,NH_3}}^b\right)}}{1 + 10^{\left(\mathrm{p}K_{\mathrm{a,COOH}}^b - \mathrm{pH}\right)} + 10^{\left(\mathrm{pH} - \mathrm{p}K_{\mathrm{a,NH_3}}^b\right)}} \tag{7}$$

and

$$\nu_f = \frac{10^{\left(\mathrm{p}K_{\mathrm{a,COOH}}^f - \mathrm{pH}\right)} - 10^{\left(\mathrm{pH} - \mathrm{p}K_{\mathrm{a,NH_3}}^f\right)}}{1 + 10^{\left(\mathrm{p}K_{\mathrm{a,COOH}}^f - \mathrm{pH}\right)} + 10^{\left(\mathrm{pH} - \mathrm{p}K_{\mathrm{a,NH_3}}^f\right)}} \tag{8}$$

A positive value of n_H indicates net proton uptake upon binding, whereas a negative value indicates net proton release. In this way, the model provides both the pH-dependent apparent binding affinity and the associated proton linkage contribution to complex formation.

5 ITC measurements

The binding affinity of selected amino acids toward CB7 was measured using a MicroCal ITC instrument in 20 mM buffer solutions at 25 °C. All experiments were performed by the same experimenter using the same instrument, and each experiment was repeated at least twice (Table S13). Individual results are summarised in Tables S14–S19.

5.1 pH-dependent ITC fitting procedure

The pH-dependent ITC data were analysed using an apparent one-proton linkage model. For each amino-acid complex, the observed dissociation constant at a given pH was fitted according to:

$$K_{D,\mathrm{obs}}(\mathrm{pH}) = K_{D,\mathrm{high}} \frac{1 + 10^{\mathrm{p}K_{I,\mathrm{free}} - \mathrm{pH}}}{1 + 10^{\mathrm{p}K_{I,\mathrm{bound}} - \mathrm{pH}}}, \tag{9}$$

where $K_{D,\mathrm{high}}$ is the limiting dissociation constant at high pH, $\mathrm{p}K_{I,\mathrm{free}}$ is the apparent acid–base constant of the free amino acid used as a fixed input parameter, and $\mathrm{p}K_{I,\mathrm{bound}}$ is the corresponding apparent value in the CB7-bound state. The fitted parameters were therefore $\log_{10} K_{D,\mathrm{high}}$ and $\mathrm{p}K_{I,\mathrm{bound}}$. The low-pH limiting dissociation constant was calculated from:

$$K_{D,\mathrm{low}} = K_{D,\mathrm{high}} 10^{\mathrm{p}K_{I,\mathrm{free}} - \mathrm{p}K_{I,\mathrm{bound}}}. \tag{10}$$

The fits were carried out by non-linear least-squares minimisation in $\log_{10} K_D$ space. The residuals were weighted using the propagated experimental uncertainty in $\log_{10} K_D$,

$$\sigma_{\log K_D} = \frac{\sigma_{K_D}}{K_D \ln 10}, \tag{11}$$

with a lower uncertainty floor of 0.03 log units. A robust soft-L1 loss function was used to reduce the influence of individual outlying points. The reported uncertainties are approximate standard errors obtained from the covariance matrix estimated from the Jacobian of the least-squares fit.

Table S12: Parameters obtained from pH-dependent ITC fitting for CB7 complexes with amino acids. Dissociation constants are given in mM. $K_{D,\mathrm{low}}$ and $K_{D,\mathrm{high}}$ denote the low- and high-pH limiting values extrapolated from the fitted linkage model rather than values measured at specific experimental pH points. Reported uncertainties are approximate standard errors obtained from the covariance matrix of the fit; uncertainties of the derived low-pH limiting values were propagated including covariance between the fitted parameters.

Ligand	$\mathrm{p}K_{I,\mathrm{free}}$	$\mathrm{p}K_{I,\mathrm{bound}}$	$\Delta\mathrm{p}K_I$	$K_{D,\mathrm{low}}$	$K_{D,\mathrm{high}}$	No. points
Phe	5.48	7.48 ± 0.09	2.00 ± 0.09	0.00127 ± 0.00016	0.126 ± 0.019	15
Tyr	5.66	7.64 ± 0.11	1.98 ± 0.11	0.592 ± 0.098	57.2 ± 7.2	8
Trp	5.94	6.60 ± 0.09	0.66 ± 0.09	0.0789 ± 0.0144	0.364 ± 0.031	9
DOPA	5.60	6.38 ± 0.13	0.78 ± 0.13	0.696 ± 0.103	4.24 ± 1.03	8
Leu	5.98	6.91 ± 0.03	0.93 ± 0.03	0.926 ± 0.035	7.80 ± 0.44	14
Met	5.75	6.85 ± 0.22	1.10 ± 0.22	0.407 ± 0.078	5.07 ± 1.79	14

Table S13: Observed pH-dependent ITC dissociation constants used for the fitting. Values of $K_{D,\mathrm{obs}}$ are given as mean ± SD in mM; n is the number of independent fitted titrations retained after data cleaning. The corresponding observed Gibbs free energies were calculated from the mean $K_{D,\mathrm{obs}}$ values using $\Delta G_{\mathrm{obs}} = RT \ln K_{D,\mathrm{obs}}$, with K_D expressed in mol L^{-1}.

Ligand	pH	$K_{D,\mathrm{obs}}$ / mM	ΔG_{obs} / kJ mol^{-1}	n
Phe	4.7	0.00139 ± 0.00004	-33.4	4
Phe	6.5	0.0155 ± 0.00021	-27.5	2
Phe	7.0	0.0459 ± 0.00219	-24.8	2
Phe	7.4	0.0369 ± 0.00075	-25.3	3
Phe	7.5	0.120 ± 0.00495	-22.4	2
Phe	8.5	0.114 ± 0.00283	-22.5	2
Tyr	4.7	0.533 ± 0.00354	-18.7	2
Tyr	6.5	5.28 ± 0.615	-13.0	2
Tyr	7.5	24.9 ± 1.48	-9.2	2
Tyr	8.5	49.4 ± 2.90	-7.5	2
Trp	4.7	0.0760 ± 0.00085	-23.5	2
Trp	6.5	0.287 ± 0.0324	-20.2	3
Trp	7.5	0.316 ± 0.00919	-20.0	2
Trp	8.5	0.357 ± 0.00707	-19.7	2
DOPA	4.7	0.794 ± 0.0127	-17.7	2
DOPA	6.5	1.49 ± 0.0354	-16.1	2
DOPA	7.5	3.72 ± 0.00707	-13.9	2
DOPA	8.5	5.13 ± 0.0424	-13.1	2
Leu	4.7	0.938 ± 0.0334	-17.3	4
Leu	6.5	3.26 ± 0.116	-14.2	3
Leu	7.0	6.31 ± 0.520	-12.6	3
Leu	7.5	6.30 ± 0.267	-12.6	4
Met	4.7	0.422 ± 0.0679	-19.3	2
Met	6.5	1.87 ± 0.0141	-15.6	2
Met	7.0	6.57 ± 1.67	-12.5	3
Met	7.5	3.62 ± 0.104	-13.9	4
Met	8.5	14.4 ± 2.54	-10.5	3

Table S14: ITC-derived binding parameters for the complexation of Phe with CB7 at different pH values.

pH	Buffer	No. titrations	K_D (μM)	ΔG_{obs} (kJ mol^{-1})	ΔH_{obs} (kJ mol^{-1})	N
4.7	acetate	2	1.37 ± 0.04	-33.5	-22.2 ± 0.8	1.00 ± 0.00
4.7	citrate	2	1.43 ± 0.01	-33.4	-23.7 ± 0.3	1.00 ± 0.00
6.5	PIPES	2	15.45 ± 0.21	-27.5	-22.9 ± 0.6	1.00 ± 0.00
7.0	phosphate	2	45.85 ± 2.19	-24.8	-14.1 ± 0.2	1.00 ± 0.00
7.4	phosphate	3	36.87 ± 0.75	-25.3	-21.4 ± 0.4	1.05 ± 0.04
7.5	TRIS	2	120.50 ± 4.95	-22.4	-21.1 ± 0.4	1.00 ± 0.00
8.5	TRIS	2	114.00 ± 2.83	-22.5	-21.6 ± 0.2	1.00 ± 0.00

Values are reported as mean ± standard deviation from independent titrations fitted using an apparent 1:1 binding model. ΔG_{obs} was calculated from the mean K_D value using $\Delta G_{\mathrm{obs}} = RT \ln K_D$, with K_D expressed in mol L^{-1}.

Table S15: ITC-derived binding parameters for the complexation of Tyr with CB7 at different pH values.

pH	Buffer	No. titrations	K_D (mM)	ΔG_{obs} (kJ mol^{-1})	ΔH_{obs} (kJ mol^{-1})
4.7	acetate	2	0.533 ± 0.004	-18.7	-1.72 ± 0.04
6.5	PIPES	2	5.28 ± 0.62	-13.0	-12.7 ± 2.1
7.5	TRIS	2	24.9 ± 1.5	-9.2	-24.6 ± 2.0
8.5	TRIS	2	49.4 ± 2.9	-7.5	-21.7 ± 1.1

Values are reported as mean ± standard deviation from independent titrations fitted using an apparent 1:1 binding model. ΔG_{obs} was calculated from the mean K_D value.

Table S16: Observed pH-dependent ITC dissociation constants for the complexation of DOPA with CB7. Values of $K_{D,\text{obs}}$ are given as mean ± SD in mM; n is the number of independent fitted titrations retained after data cleaning. The corresponding observed Gibbs free energies were calculated from the mean $K_{D,\text{obs}}$ values using $\Delta G_{\text{obs}} = RT \ln K_{D,\text{obs}}$, with K_D expressed in mol L^{-1}.

pH	Buffer	$K_{D,\text{obs}}$ / mM	ΔG_{obs} / kJ mol^{-1}	n
4.7	acetate	0.794 ± 0.0127	-17.7	2
6.5	PIPES	1.49 ± 0.0354	-16.1	2
7.5	TRIS	3.72 ± 0.00707	-13.9	2
8.5	TRIS	5.13 ± 0.0424	-13.1	2

Table S17: Observed pH-dependent ITC dissociation constants for the complexation of L-Trp with CB7. Values of $K_{D,\text{obs}}$ are given as mean ± SD in mM; n is the number of independent fitted titrations retained after data cleaning. The corresponding observed Gibbs free energies were calculated from the mean $K_{D,\text{obs}}$ values using $\Delta G_{\text{obs}} = RT \ln K_{D,\text{obs}}$, with K_D expressed in mol L^{-1}.

pH	Buffer	$K_{D,\text{obs}}$ / mM	ΔG_{obs} / kJ mol^{-1}	n
4.7	acetate	0.0760 ± 0.00085	-23.5	2
6.5	PIPES	0.287 ± 0.0324	-20.2	3
7.5	PIPES	0.316 ± 0.00919	-20.0	2
8.5	TRIS	0.357 ± 0.00707	-19.7	2

Table S18: Observed pH-dependent ITC dissociation constants for the complexation of Met with CB7. Values of $K_{D,\text{obs}}$ are given as mean ± SD in mM; n is the number of independent fitted titrations retained after data cleaning. The corresponding observed Gibbs free energies were calculated from the mean $K_{D,\text{obs}}$ values using $\Delta G_{\text{obs}} = RT \ln K_{D,\text{obs}}$, with K_D expressed in mol L^{-1}.

pH	Buffer	$K_{D,\text{obs}}$ / mM	ΔG_{obs} / kJ mol^{-1}	n
4.7	acetate	0.422 ± 0.0679	-19.3	2
6.5	PIPES	1.87 ± 0.0141	-15.6	2
7.0	phosphate	6.57 ± 1.67	-12.5	3
7.5	PIPES	3.62 ± 0.104	-13.9	4
8.5	TRIS	14.4 ± 2.54	-10.5	3

Table S19: Observed pH-dependent ITC dissociation constants for the complexation of L-Leu with CB7. Values of $K_{D,\text{obs}}$ are given as mean ± SD in mM; n is the number of independent fitted titrations retained after data cleaning. The corresponding observed Gibbs free energies were calculated from the mean $K_{D,\text{obs}}$ values using $\Delta G_{\text{obs}} = RT \ln K_{D,\text{obs}}$, with K_D expressed in mol L^{-1}.

pH	Buffer	$K_{D,\text{obs}}$ / mM	ΔG_{obs} / kJ mol^{-1}	n
4.7	acetate	0.938 ± 0.0334	-17.3	4
6.5	PIPES	3.26 ± 0.116	-14.2	3
7.0	phosphate	6.31 ± 0.520	-12.6	3
7.5	PIPES	6.32 ± 0.460	-12.6	2
7.5	TRIS	6.28 ± 0.000	-12.6	2

6 Molecular Dynamics Simulations

MD simulations of the CB7 complexes with Phe, Tyr, DOPA, and Trp were performed with `pmemd.MPI` in AMBER22.[S4] CB7 and the ligands were parameterized with GAFF2, and RESP charges were derived from electrostatic potentials computed with Gaussian16 at the B3LYP/6-311+G* level. Periodic boundary conditions were applied; the simulation box contained the host, the guest, and 2000 TIP3P water molecules. Simulations were carried out at 298.15 K using a Langevin thermostat and at 1 bar using a Monte Carlo barostat for production. Long-range electrostatics were treated with PME, and a 12 Å cutoff was used for non-bonded interactions.

For Phe@CB7 and Tyr@CB7, additional independent replicas were prepared to test the effect of the initial hydration state of the CB7 cavity. Two sets of starting structures were generated: one containing a water molecule in the CB7 cavity close to the ammonium group, denoted HOH, and one without an initial cavity water molecule, denoted nHOH. For each hydration condition, three independent replicas were run using different initial velocity seeds. These simulations were used to assess whether cavity-water-containing and cavity-water-free starting structures converge to similar hydration-state populations, or whether the sampled populations depend on the initial cavity hydration.

The hydration-state classification used a core-first, mutually exclusive assignment. The main MD-derived states were defined as follows: the *core-contact* state, in which a water molecule occupies the central part of the CB7 cavity and directly contacts the ammonium group of the guest; the *core-no-contact* state, in which a water molecule is present in the central part of the cavity, typically on the opposite side relative to the ammonium group, and therefore does not form a direct water–NH_3^+ contact; the *portal-only* state, in which water is localized at the portal region in the absence of core water; and the *dry-cavity* state, in which neither core water nor portal water is present within the analysed hydration regions. Because the assignment is core-first, additional portal water may coexist with the *core-contact* and *core-no-contact* states; therefore, *portal-only* denotes portal hydration only for frames without core water. The full classification also included low-population states, `core_no_contact_same`, `portal_contact_only`, and `core_no_contact_ambiguous`. The `core_no_contact_same` state corresponds to a rare same-side core-water configuration without direct water–NH_3^+ contact, whereas the remaining low-population states represent rare portal-contact or ambiguous core-water assignments. These low-population states were omitted from the summary tables for clarity because they account only for a minor fraction of the trajectory and do not affect the overall interpretation.

To quantify these differences, the binding poses were compared using a common CB7-based geometric reference frame (Fig. S6). The reference frame is defined by the centroids of the two CB7 portals and of the CB7 mid-plane, with the portal1→portal2 vector defining the axial coordinate. Portal 1 was assigned as the portal interacting with the ammonium group. The projected positions of the ammonium nitrogen N_α and the ligand center of geometry (COG)

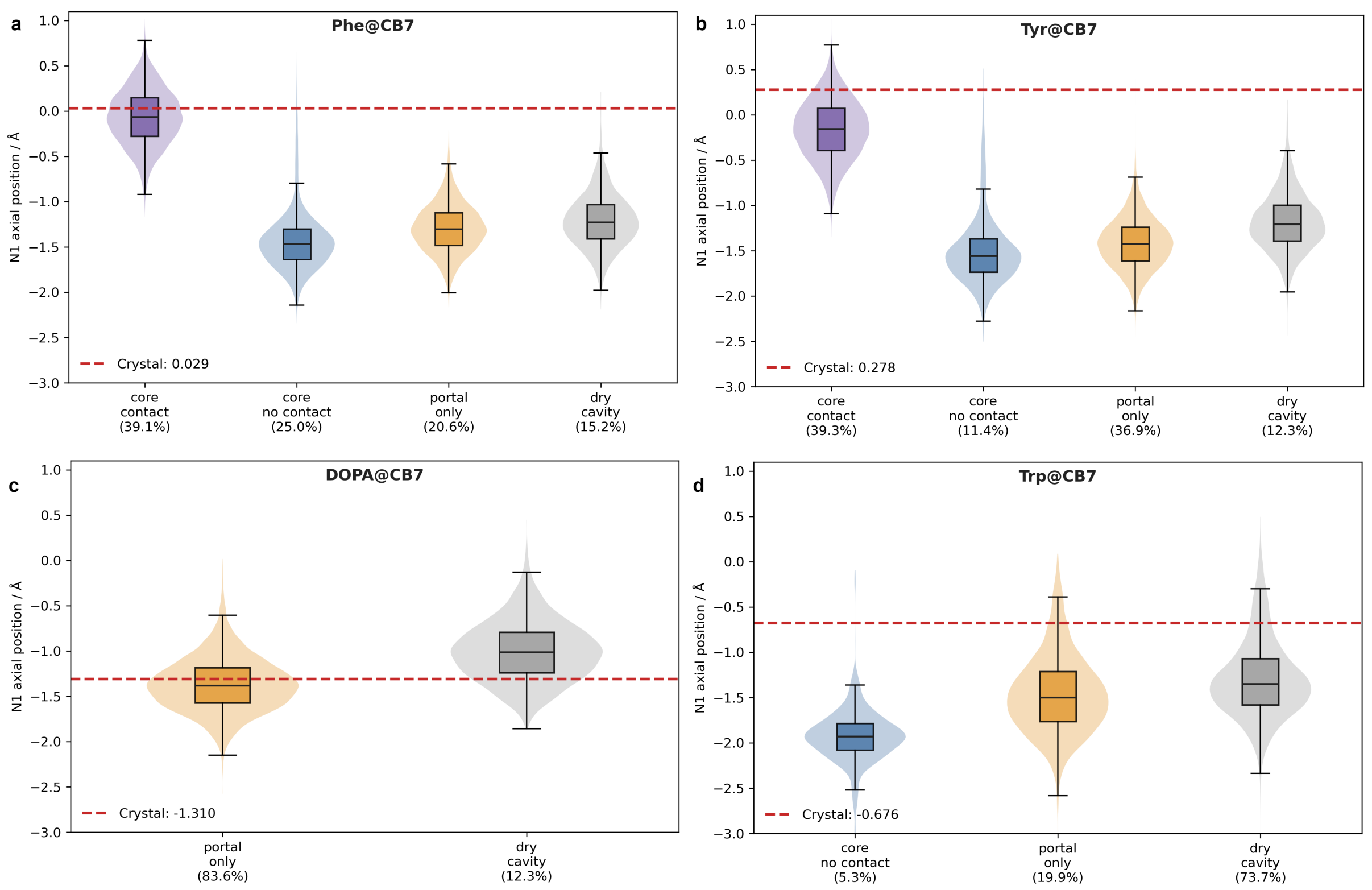

Figure S8: State-resolved distributions of N_α axial position for aromatic amino acid complexes with CB7 obtained from MD simulations. The four hydration states are shown as violin plots with embedded box plots, and the corresponding optimized XRD-derived reference values are indicated by red dashed lines. (a) Phe@CB7, (b) Tyr@CB7, (c) DOPA@CB7, and (d) Trp@CB7. State populations are given below the x-axis labels.

were measured along this axis from portal 1. In this convention, negative axial positions indicate displacement outside the CB7 cavity, whereas positive values indicate displacement deeper into the cavity. For aromatic guests, the acute ring-plane angle was defined as the acute angle between the aromatic ring plane and the CB7 mid-plane (Tables S4–S5).

For Tyr@CB7, the *portal-only* and *core-contact* states are the two dominant states in all replicas. Compared with Phe@CB7, Tyr samples a larger fraction of the *portal-only* state in several runs. Within each hydration state, however, the state-resolved geometries are highly similar across replicas. This indicates that the differences between replicas arise mainly from changes in hydration-state populations rather than from changes in the geometry of a given state.

DOPA@CB7 shows a different hydration-state distribution. The *portal-only* state dominates, accounting for 83.61% of the analysed frames, whereas the *core-contact* state accounts for only 3.17%. The remaining major population corresponds to the *dry-cavity* state.

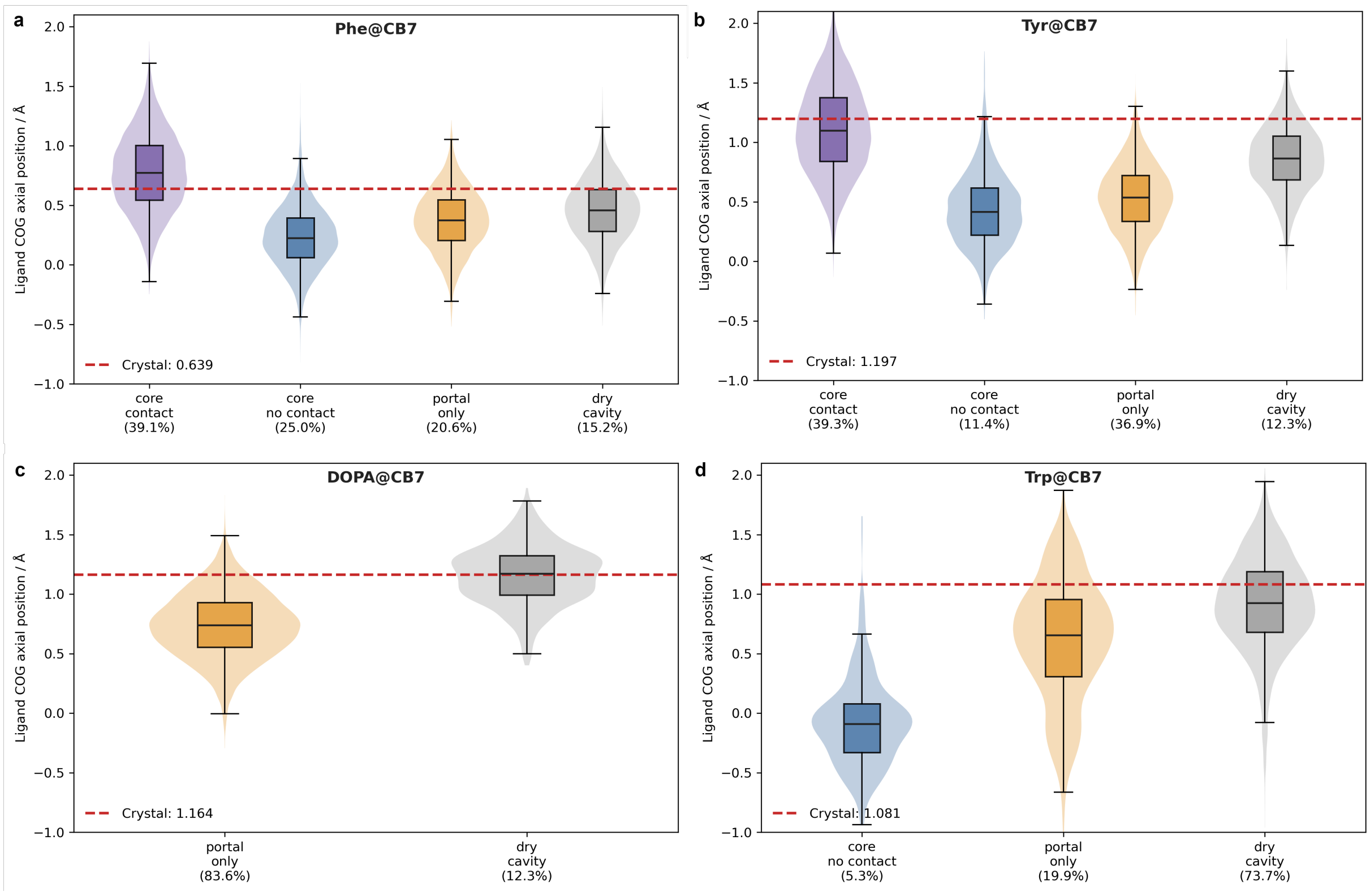

Figure S9: State-resolved distributions of ligand COG axial position for aromatic amino acid complexes with CB7 obtained from MD simulations. The four hydration states are shown as violin plots with embedded box plots, and the corresponding optimized XRD-derived reference values are indicated by red dashed lines. (a) Phe@CB7, (b) Tyr@CB7, (c) DOPA@CB7, and (d) Trp@CB7. State populations are given below the x-axis labels.

Table S20: Trajectory-averaged water statistics for the amino acid@CB7 replicas. Reported values were calculated over all analysed frames and summarize the average hydration of the core, portal, and bridge regions.

Guest	System	Seed	Mean core waters	Mean portal waters	Mean bridge waters	Mean core-contact waters
Phe	HOH	s1	0.687	0.793	1.451	0.392
Phe	HOH	s3	0.645	0.800	1.481	0.350
Phe	HOH	s4	0.730	0.791	1.437	0.453
Phe	nHOH	s1	0.625	0.803	1.504	0.314
Phe	nHOH	s3	0.753	0.783	1.405	0.489
Phe	nHOH	s4	0.565	0.826	1.548	0.230
Tyr	HOH	s1	0.597	0.861	1.946	0.393
Tyr	HOH	s2	0.619	0.873	1.930	0.403
Tyr	HOH	s3	0.489	0.907	1.986	0.283
Tyr	nHOH	s1	0.537	0.892	1.968	0.329
Tyr	nHOH	s2	0.403	0.949	2.004	0.217
Tyr	nHOH	s3	0.624	0.843	1.942	0.391
DOPA	–	–	0.044	0.966	2.095	0.032
Trp	–	–	0.059	0.274	1.247	0.005

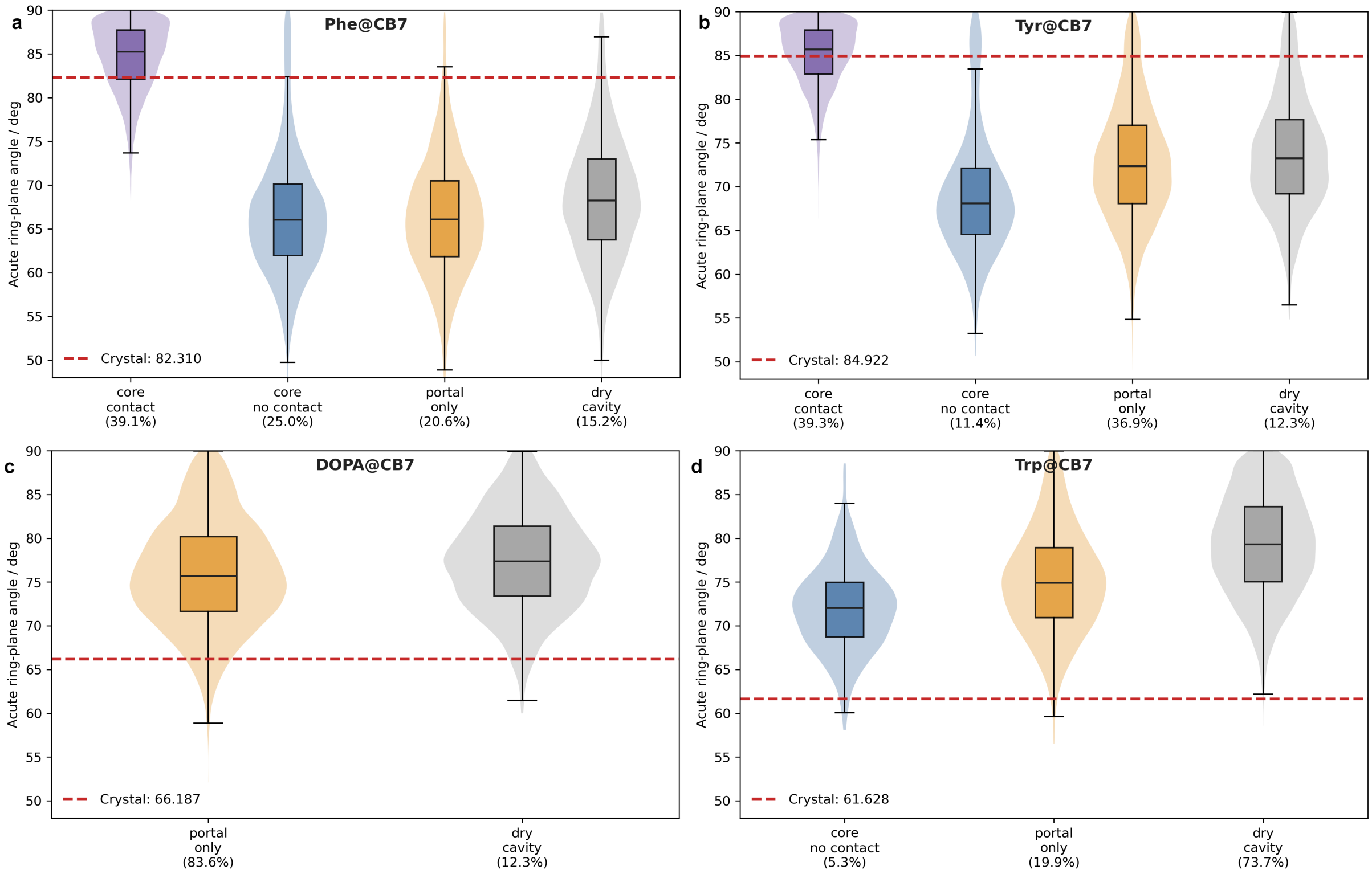

Figure S10: State-resolved distributions of acute ring-plane angle for aromatic amino acid complexes with CB7 obtained from MD simulations. The four hydration states are shown as violin plots with embedded box plots, and the corresponding optimized XRD-derived reference values are indicated by red dashed lines. (a) Phe@CB7, (b) Tyr@CB7, (c) DOPA@CB7, and (d) Trp@CB7. State populations are given below the x-axis labels.

Table S21: Water occupancy and residence-event analysis for the core and portal hydration regions in Phe@CB7 and Tyr@CB7. Occ. is the percentage of analysed MD frames in which at least one water oxygen atom is present in the region. N_{ev} is the number of continuous residence events identified from the per-frame hydration assignment. Residence times are given as median, mean, and maximum values. Saved trajectory frames were spaced by 10 ps.

Complex	Region	Occ. / %	N_{ev}	t_{med} / ns	t_{mean} / ns	t_{max} / ns
Phe@CB7	core	64.1	2776	0.02	0.074	9.47
Phe@CB7	portal	65.9	16525	0.01	0.014	0.13
Tyr@CB7	core	50.7	2244	0.01	0.080	15.08
Tyr@CB7	portal	70.5	15627	0.01	0.017	0.12

Table S22: Summary of the full 300 ns MD analyses for the Phe@CB7 replicas initiated with a cavity water molecule (HOH) and without cavity water (nHOH). The table compares the mean axial position of the ammonium nitrogen (N_α), mean axial position of the ligand center of geometry (COG), mean acute angle between the aromatic ring plane and the CB7 mid-plane, and the populations of the major water/positioning states identified in the trajectory analysis.

System	Seed	N_α axial mean (Å)	Ligand COG axial mean (Å)	Ring angle mean (°)	*core-contact* (%)	*portal-only* (%)	*dry-cavity* (%)
HOH	s1	-0.836	0.509	73.892	39.14	20.61	15.24
HOH	s3	-0.886	0.489	73.211	34.92	22.94	16.58
HOH	s4	-0.752	0.537	75.132	45.25	18.68	13.46
nHOH	s1	-0.934	0.474	72.512	31.41	23.96	17.16
nHOH	s3	-0.704	0.554	75.766	48.85	17.46	12.53
nHOH	s4	-1.047	0.432	70.986	23.02	27.09	19.28

Table S23: State-resolved geometry for the Phe@CB7 replicas initiated with a cavity water molecule (HOH). Only the major MD-derived hydration states are shown: *core-contact*, *core-no-contact*, *portal-only*, and *dry-cavity*, as defined in the Molecular Dynamics section. Reported values are mean ± standard deviation calculated over frames assigned to a given state. Low-population states were omitted for clarity.

State	Seed	Population (%)	N_α axial position (Å)	Ligand COG axial position (Å)	Acute ring-plane angle (°)	$N_\alpha \cdots O_w$ (Å)
core-contact	s1	39.14	-0.068 ± 0.311	0.778 ± 0.325	84.565 ± 3.955	2.984 ± 0.156
	s3	34.92	-0.062 ± 0.307	0.774 ± 0.323	84.638 ± 3.898	2.982 ± 0.154
	s4	45.25	-0.063 ± 0.311	0.779 ± 0.324	84.654 ± 3.884	2.984 ± 0.155
core-no-contact	s1	23.54	-1.491 ± 0.233	0.208 ± 0.237	65.540 ± 5.651	6.355 ± 0.321
	s3	24.16	-1.491 ± 0.236	0.207 ± 0.237	65.470 ± 5.598	6.354 ± 0.319
	s4	20.93	-1.490 ± 0.237	0.204 ± 0.240	65.470 ± 5.583	6.352 ± 0.318
portal-only	s1	20.61	-1.295 ± 0.272	0.372 ± 0.254	66.351 ± 6.737	–
	s3	22.94	-1.292 ± 0.270	0.372 ± 0.251	66.443 ± 6.740	–
	s4	18.68	-1.296 ± 0.276	0.373 ± 0.256	66.560 ± 6.701	–
dry-cavity	s1	15.24	-1.212 ± 0.301	0.458 ± 0.264	68.605 ± 7.080	–
	s3	16.58	-1.209 ± 0.301	0.459 ± 0.264	68.870 ± 7.049	–
	s4	13.46	-1.201 ± 0.309	0.466 ± 0.268	68.883 ± 7.209	–

Table S24: State-resolved geometry for the Phe@CB7 replicas initiated without a cavity water molecule (nHOH). Only the major MD-derived hydration states are shown: *core-contact*, *core-no-contact*, *portal-only*, and *dry-cavity*, as defined in the Molecular Dynamics section. Reported values are mean ± standard deviation calculated over frames assigned to a given state. Low-population states were omitted for clarity.

State	Seed	Population (%)	N_α axial position (Å)	Ligand COG axial position (Å)	Acute ring-plane angle (°)	$N_\alpha \cdots O_w$ (Å)
core-contact	s1	31.41	-0.060 ± 0.309	0.783 ± 0.325	84.608 ± 3.885	2.980 ± 0.155
	s3	48.85	-0.060 ± 0.310	0.781 ± 0.325	84.655 ± 3.898	2.980 ± 0.155
	s4	23.02	-0.066 ± 0.306	0.776 ± 0.322	84.545 ± 3.923	2.981 ± 0.155
core-no-contact	s1	26.27	-1.494 ± 0.239	0.204 ± 0.242	65.477 ± 5.637	6.357 ± 0.322
	s3	19.35	-1.498 ± 0.236	0.202 ± 0.235	65.269 ± 5.629	6.357 ± 0.320
	s4	29.77	-1.495 ± 0.237	0.202 ± 0.241	65.600 ± 5.668	6.358 ± 0.323
portal-only	s1	23.96	-1.292 ± 0.277	0.370 ± 0.254	66.525 ± 6.714	–
	s3	17.46	-1.288 ± 0.277	0.376 ± 0.258	66.545 ± 6.566	–
	s4	27.09	-1.292 ± 0.273	0.371 ± 0.252	66.601 ± 6.657	–
dry-cavity	s1	17.16	-1.209 ± 0.302	0.460 ± 0.265	68.714 ± 7.033	–
	s3	12.53	-1.204 ± 0.309	0.459 ± 0.267	68.966 ± 7.166	–
	s4	19.28	-1.209 ± 0.305	0.459 ± 0.267	68.687 ± 7.054	–

Table S25: Bridge-summary analysis for the Phe@CB7 replicas initiated with a cavity water molecule (HOH). Only the major MD-derived hydration states are shown: *core-contact*, *core-no-contact*, *portal-only*, and *dry-cavity*, as defined in the Molecular Dynamics section. For each state, the table reports the number of frames, population, mean total number of bridge waters, fraction of frames containing at least one bridge water, mean numbers of UU and UV hydrogen bonds, mean number of core waters, and mean number of core-contact waters. Low-population states were omitted for clarity.

State	Seed	n_{frames}	Population (%)	Mean bridge waters	Frames with bridge water (%)	Mean HB_{UU}	Mean HB_{UV}	Mean core waters	Mean core-contact waters
core-contact	s1	11741	39.14	1.138	75.16	0.610	15.812	1.110	1.001
core-no-contact	s1	7061	23.54	1.614	87.96	0.506	16.244	1.011	0.000
portal-only	s1	6183	20.61	1.691	89.18	0.443	16.081	0.000	0.000
dry-cavity	s1	4571	15.24	1.709	89.98	0.408	15.884	0.000	0.000
core-contact	s3	10477	34.92	1.146	75.55	0.610	15.832	1.106	1.002
core-no-contact	s3	7249	24.16	1.626	88.18	0.496	16.251	1.009	0.000
portal-only	s3	6883	22.94	1.704	89.70	0.446	16.071	0.000	0.000
dry-cavity	s3	4975	16.58	1.698	89.99	0.411	15.864	0.000	0.000
core-contact	s4	13575	45.25	1.164	76.38	0.608	15.838	1.108	1.001
core-no-contact	s4	6279	20.93	1.619	88.53	0.496	16.324	1.010	0.000
portal-only	s4	5603	18.68	1.704	89.36	0.432	16.148	0.000	0.000
dry-cavity	s4	4039	13.46	1.736	90.05	0.410	15.891	0.000	0.000

Table S26: Bridge-summary analysis for the Phe@CB7 replicas initiated without a cavity water molecule (nHOH). Only the major states are shown: *core-contact*, *core-no-contact*, *portal-only*, and *dry-cavity*, as defined in the Molecular Dynamics section. For each state, the table reports the number of frames, population, mean total number of bridge waters, fraction of frames containing at least one bridge water, mean numbers of UU and UV hydrogen bonds, mean number of core waters, and mean number of core-contact waters. Low-population states were omitted for clarity.

State	Seed	n_{frames}	Population (%)	Mean bridge waters	Frames with bridge water (%)	Mean HB_{UU}	Mean HB_{UV}	Mean core waters	Mean core-contact waters
core-contact	s1	9423	31.41	1.160	75.68	0.620	15.809	1.105	1.001
core-no-contact	s1	7880	26.27	1.612	88.13	0.518	16.300	1.012	0.000
portal-only	s1	7188	23.96	1.703	89.71	0.428	16.128	0.000	0.000
dry-cavity	s1	5147	17.16	1.713	89.92	0.414	15.909	0.000	0.000
core-contact	s3	14654	48.85	1.155	75.95	0.619	15.853	1.105	1.001
core-no-contact	s3	5805	19.35	1.609	88.29	0.506	16.275	1.008	0.000
portal-only	s3	5238	17.46	1.697	89.73	0.440	16.111	0.000	0.000
dry-cavity	s3	3760	12.53	1.698	88.78	0.424	15.812	0.000	0.000
core-contact	s4	6906	23.02	1.140	75.28	0.614	15.888	1.109	1.001
core-no-contact	s4	8930	29.77	1.613	88.24	0.509	16.326	1.010	0.000
portal-only	s4	8127	27.09	1.695	89.23	0.439	16.119	0.000	0.000
dry-cavity	s4	5783	19.28	1.747	90.80	0.407	15.869	0.000	0.000

Table S27: Per-frame water statistics for the Phe@CB7 trajectories. Reported values were calculated over the full 300 ns trajectories (30 000 analysed frames per replica). The table summarizes the mean numbers of core and portal waters, the mean number of core-contact waters, and the fractions of frames containing core or portal waters.

System	Seed	Mean core waters	Mean portal waters	Mean core-contact waters	Frames with core water (%)	Frames with ≥ 2 core waters (%)	Frames with portal water (%)	Frames with ≥ 2 portal waters (%)
HOH	s1	0.687	0.793	0.392	64.12	4.58	65.87	13.12
HOH	s3	0.645	0.800	0.350	60.46	3.97	66.57	13.08
HOH	s4	0.730	0.791	0.453	67.85	5.18	65.56	13.17
nHOH	s1	0.625	0.803	0.314	58.83	3.61	66.58	13.32
nHOH	s3	0.753	0.783	0.489	70.00	5.32	64.75	13.06
nHOH	s4	0.565	0.826	0.230	53.62	2.82	68.47	13.73

Table S28: Summary of the full 300 ns MD analyses for the Tyr@CB7 replicas initiated with a cavity water molecule (HOH) and without cavity water (nHOH). The table compares the mean axial position of the ammonium nitrogen (N_α), mean axial position of the ligand center of geometry (COG), mean acute angle between the aromatic ring plane and the CB7 mid-plane, and the populations of the major water/positioning states identified in the trajectory analysis.

System	Seed	N_α axial mean (Å)	Ligand COG axial mean (Å)	Ring angle mean (°)	*core-contact* (%)	*portal-only* (%)	*dry-cavity* (%)
HOH	s1	-0.904	0.788	77.308	39.29	36.90	12.34
HOH	s2	-0.898	0.789	77.398	40.24	35.73	11.69
HOH	s3	-1.047	0.725	75.858	28.26	43.47	14.37
nHOH	s1	-0.989	0.752	76.380	32.84	40.81	12.76
nHOH	s2	-1.126	0.694	74.992	21.65	48.87	15.79
nHOH	s3	-0.907	0.787	77.255	39.13	37.62	12.09

Table S29: State-resolved geometry for the Tyr@CB7 replicas initiated with a cavity water molecule (HOH). Only the major MD-derived hydration states are shown: *core-contact*, *core-no-contact*, *portal-only*, and *dry-cavity*, as defined in the Molecular Dynamics section. Reported values are mean ± standard deviation calculated over frames assigned to a given state. Low-population states were omitted for clarity.

State	Seed	Population (%)	N_α axial position (Å)	Ligand COG axial position (Å)	Acute ring-plane angle (°)	$N_\alpha \cdots O_w$ (Å)
core-contact	s1	39.29	-0.161 ± 0.335	1.107 ± 0.372	85.057 ± 3.576	2.953 ± 0.146
	s2	40.24	-0.163 ± 0.334	1.104 ± 0.369	85.080 ± 3.490	2.953 ± 0.148
	s3	28.26	-0.166 ± 0.335	1.104 ± 0.370	85.072 ± 3.558	2.950 ± 0.147
core-no-contact	s1	10.32	-1.593 ± 0.241	0.392 ± 0.266	67.510 ± 4.995	6.396 ± 0.329
	s2	11.20	-1.610 ± 0.239	0.380 ± 0.263	67.466 ± 4.986	6.404 ± 0.338
	s3	13.10	-1.607 ± 0.239	0.379 ± 0.265	67.563 ± 5.026	6.404 ± 0.336
portal-only	s1	36.90	-1.125 ± 0.359	0.764 ± 0.370	78.936 ± 5.634	–
	s2	35.73	-1.117 ± 0.359	0.771 ± 0.366	79.124 ± 5.537	–
	s3	43.47	-1.117 ± 0.354	0.758 ± 0.365	79.014 ± 5.589	–
dry-cavity	s1	12.34	-1.346 ± 0.302	0.625 ± 0.314	72.556 ± 6.069	–
	s2	11.69	-1.344 ± 0.306	0.628 ± 0.313	72.650 ± 6.096	–
	s3	14.37	-1.345 ± 0.304	0.626 ± 0.315	72.457 ± 6.116	–

Table S30: State-resolved geometry for the Tyr@CB7 replicas initiated without a cavity water molecule (nHOH). Only the major MD-derived hydration states are shown: *core-contact*, *core-no-contact*, *portal-only*, and *dry-cavity*, as defined in the Molecular Dynamics section. Reported values are mean ± standard deviation calculated over frames assigned to a given state. Low-population states were omitted for clarity.

State	Seed	Population (%)	N_α axial position (Å)	Ligand COG axial position (Å)	Acute ring-plane angle (°)	$N_\alpha \cdots O_w$ (Å)
core-contact	s1	32.84	-0.155 ± 0.334	1.119 ± 0.367	84.983 ± 3.613	2.953 ± 0.147
	s2	21.65	-0.158 ± 0.335	1.119 ± 0.368	85.039 ± 3.531	2.957 ± 0.151
	s3	39.13	-0.162 ± 0.337	1.110 ± 0.373	84.956 ± 3.593	2.954 ± 0.147
core-no-contact	s1	12.60	-1.610 ± 0.246	0.382 ± 0.267	67.513 ± 5.028	6.397 ± 0.349
	s2	13.08	-1.600 ± 0.238	0.377 ± 0.270	67.544 ± 5.031	6.402 ± 0.339
	s3	10.07	-1.609 ± 0.240	0.375 ± 0.269	67.422 ± 5.043	6.398 ± 0.339
portal-only	s1	40.81	-1.115 ± 0.354	0.767 ± 0.366	78.918 ± 5.510	–
	s2	48.87	-1.114 ± 0.355	0.765 ± 0.368	78.904 ± 5.553	–
	s3	37.62	-1.114 ± 0.357	0.774 ± 0.370	78.918 ± 5.559	–
dry-cavity	s1	12.76	-1.346 ± 0.312	0.631 ± 0.319	72.604 ± 6.196	–
	s2	15.79	-1.338 ± 0.313	0.627 ± 0.321	72.463 ± 6.143	–
	s3	12.09	-1.337 ± 0.314	0.635 ± 0.317	72.626 ± 6.211	–

Table S31: Bridge-summary analysis for the Tyr@CB7 replicas initiated with a cavity water molecule (HOH). Only the major states are shown: *core-contact*, *core-no-contact*, *portal-only*, and *dry-cavity*, as defined in the Molecular Dynamics section. For each state, the table reports the number of frames, population, mean total number of bridge waters, fraction of frames containing at least one bridge water, mean numbers of UU and UV hydrogen bonds, mean number of core waters, and mean number of core-contact waters. Low-population states were omitted for clarity.

State	Seed	n_{frames}	Population (%)	Mean bridge waters	Frames with bridge water (%)	Mean HB_{UU}	Mean HB_{UV}	Mean core waters	Mean core-contact waters
core-contact	s1	11787	39.29	1.762	87.99	0.772	16.759	1.217	1.001
core-no-contact	s1	3095	10.32	1.848	91.11	0.668	17.332	1.029	0.000
portal-only	s1	11071	36.90	2.144	94.50	0.553	17.480	0.000	0.000
dry-cavity	s1	3703	12.34	2.049	92.33	0.667	16.423	0.000	0.000
core-contact	s2	12071	40.24	1.760	88.32	0.766	16.796	1.220	1.001
core-no-contact	s2	3360	11.20	1.841	90.68	0.676	17.418	1.034	0.000
portal-only	s2	10719	35.73	2.127	94.09	0.536	17.478	0.000	0.000
dry-cavity	s2	3506	11.69	2.008	93.15	0.686	16.309	0.000	0.000
core-contact	s3	8479	28.26	1.770	87.77	0.766	16.817	1.225	1.000
core-no-contact	s3	3929	13.10	1.843	91.91	0.657	17.378	1.025	0.000
portal-only	s3	13042	43.47	2.155	94.53	0.535	17.447	0.000	0.000
dry-cavity	s3	4311	14.37	2.039	92.69	0.705	16.340	0.000	0.000

Table S32: Bridge-summary analysis for the Tyr@CB7 replicas initiated without a cavity water molecule (nHOH). Only the major states are shown: *core-contact*, *core-no-contact*, *portal-only*, and *dry-cavity*, as defined in the Molecular Dynamics section. For each state, the table reports the number of frames, population, mean total number of bridge waters, fraction of frames containing at least one bridge water, mean numbers of UU and UV hydrogen bonds, mean number of core waters, and mean number of *core-contact* waters. Low-population states were omitted for clarity.

State	Seed	n_{frames}	Population (%)	Mean bridge waters	Frames with bridge water (%)	Mean HB_{UU}	Mean HB_{UV}	Mean core waters	Mean core-contact waters
core-contact	s1	9851	32.84	1.776	88.24	0.764	16.731	1.207	1.000
core-no-contact	s1	3781	12.60	1.839	90.85	0.668	17.469	1.030	0.000
portal-only	s1	12244	40.81	2.157	94.45	0.548	17.488	0.000	0.000
dry-cavity	s1	3829	12.76	1.996	93.00	0.672	16.317	0.000	0.000
core-contact	s2	6494	21.65	1.740	87.70	0.775	16.740	1.211	1.000
core-no-contact	s2	3924	13.08	1.833	90.80	0.667	17.400	1.028	0.000
portal-only	s2	14662	48.87	2.157	94.08	0.534	17.510	0.000	0.000
dry-cavity	s2	4736	15.79	2.045	92.97	0.667	16.397	0.000	0.000
core-contact	s3	11738	39.13	1.761	88.03	0.773	16.751	1.212	1.001
core-no-contact	s3	3020	10.07	1.845	91.52	0.663	17.437	1.034	0.000
portal-only	s3	11286	37.62	2.133	94.21	0.530	17.465	0.000	0.000
dry-cavity	s3	3626	12.09	2.039	93.27	0.662	16.368	0.000	0.000

Table S33: Per-frame water statistics for the Tyr@CB7 trajectories. Reported values were calculated over the full 300 ns trajectories (30 000 analysed frames per replica). The table summarizes the mean numbers of core and portal waters, the mean number of core-contact waters, and the fractions of frames containing core or portal waters.

System	Seed	Mean core waters	Mean portal waters	Mean core-contact waters	Frames with core water (%)	Frames with ≥2 core waters (%)	Frames with portal water (%)	Frames with ≥2 portal waters (%)
HOH	s1	0.597	0.861	0.393	50.73	8.98	70.45	15.28
HOH	s2	0.619	0.873	0.403	52.58	9.29	70.93	15.96
HOH	s3	0.489	0.907	0.283	42.15	6.75	73.21	17.04
nHOH	s1	0.537	0.892	0.329	46.42	7.26	72.46	16.30
nHOH	s2	0.403	0.949	0.217	35.33	4.98	75.46	18.14
nHOH	s3	0.624	0.843	0.391	53.59	8.82	69.08	14.69

Table S34: Summary of the full MD analysis for DOPA@CB7. The table compares the mean axial position of the ammonium nitrogen (N_α), mean axial position of the ligand center of geometry (COG), mean acute angle between the aromatic ring plane and the CB7 mid-plane, and the populations of the major water/positioning states identified in the trajectory analysis.

System	N_α axial mean (Å)	Ligand COG axial mean (Å)	Ring angle mean (°)	*core-contact* (%)	*portal-only* (%)	*dry-cavity* (%)
DOPA	-1.284	0.809	76.401	3.17	83.61	12.34

Table S35: State-resolved geometry for DOPA@CB7. Only the major MD-derived hydration states are shown: *core-contact*, *portal-only*, and *dry-cavity*, as defined in the Molecular Dynamics section. Reported values are mean ± standard deviation calculated over frames assigned to a given state. Low-population states were omitted for clarity.

State	Population (%)	N_α axial position (Å)	Ligand COG axial position (Å)	Acute ring-plane angle (°)	$N_\alpha \cdots O_w$ (Å)
core-contact	3.17	-0.130 ± 0.324	1.310 ± 0.328	84.988 ± 3.999	2.989 ± 0.161
portal-only	83.61	-1.368 ± 0.301	0.740 ± 0.276	75.978 ± 6.154	–
dry-cavity	12.34	-1.003 ± 0.336	1.161 ± 0.250	77.372 ± 5.646	–

Table S36: Bridge-summary analysis for DOPA@CB7. Only the major states are shown: *core-contact*, *portal-only*, and *dry-cavity*. For each state, the table reports the number of frames, population, mean total number of bridge waters, fraction of frames containing at least one bridge water, mean numbers of UU and UV hydrogen bonds, mean number of core waters, and mean number of core-contact waters. Low-population states were omitted for clarity.

State	n_{frames}	Population (%)	Mean bridge waters	Frames with bridge water (%)	Mean HB_{UU}	Mean HB_{UV}	Mean core waters	Mean core-contact waters
core-contact	317	3.17	1.817	88.64	0.808	16.896	1.132	1.000
portal-only	8361	83.61	2.149	95.50	0.383	17.354	0.000	0.000
dry-cavity	1234	12.34	2.082	92.79	0.474	16.372	0.000	0.000

Table S37: Summary of the full MD analysis for Trp@CB7. The table compares the mean axial position of the ammonium nitrogen (N_α), mean axial position of the ligand center of geometry (COG), mean acute angle between the aromatic ring plane and the CB7 mid-plane, and the populations of the major water/positioning states identified in the trajectory analysis.

System	N_α axial mean (Å)	Ligand COG axial mean (Å)	Ring angle mean (°)	*core-contact* (%)	*portal-only* (%)	*dry-cavity* (%)
Trp	-1.347	0.811	77.998	0.51	19.91	73.74

Table S38: State-resolved geometry for Trp@CB7. Only the major MD-derived hydration states are shown: *core-contact*, *portal-only*, and *dry-cavity*, as defined in the Molecular Dynamics section. Reported values are mean ± standard deviation calculated over frames assigned to a given state. Low-population states were omitted for clarity, except for *core-contact*, which is included because it represents the direct water–ammonium contact state.

State	Population (%)	N_α axial position (Å)	Ligand COG axial position (Å)	Acute ring-plane angle (°)	$N_\alpha \cdots O_w$ (Å)
core-contact	0.51	0.059 ± 0.342	1.547 ± 0.246	82.238 ± 3.993	2.979 ± 0.169
portal-only	19.91	-1.464 ± 0.452	0.612 ± 0.509	75.104 ± 5.946	–
dry-cavity	73.74	-1.290 ± 0.437	0.919 ± 0.401	79.197 ± 5.798	–

Table S39: Bridge-summary analysis for Trp@CB7. Only the major states are shown: *core-contact*, *portal-only*, and *dry-cavity*. For each state, the table reports the number of frames, population, mean total number of bridge waters, fraction of frames containing at least one bridge water, mean numbers of UU and UV hydrogen bonds, mean number of core waters, and mean number of core-contact waters. Low-population states were omitted for clarity, except for *core-contact*, which is included because it represents the direct water–ammonium contact state.

State	n_{frames}	Population (%)	Mean bridge waters	Frames with bridge water (%)	Mean HB_{UU}	Mean HB_{UV}	Mean core waters	Mean core-contact waters
core-contact	51	0.51	1.059	74.51	0.647	14.922	1.000	1.000
portal-only	1991	19.91	1.197	76.80	0.907	15.815	0.000	0.000
dry-cavity	7374	73.74	1.293	80.76	0.798	15.554	0.000	0.000

7 Interaction Energy Calculations

The starting geometries for all calculations were constructed as dimeric, trimeric, or tetrameric molecular clusters extracted directly from the corresponding X-ray diffraction crystal structures. Quantum-chemical calculations were performed with ORCA 6.1.[S5, S6] These optimized XRD-derived geometries were used as the reference values indicated by red dashed lines in the state-resolved MD geometry plots.

Table S40: Geometric descriptors for optimized CB7–amino acid complexes derived from crystal structures. One optimized geometry is shown for each guest/state. N_α and ligand COG positions were measured along the portal 1→portal 2 axis; the ring angle is given relative to the CB7 mid-plane.

Guest	State	N_α ax. / Å	COG ax. / Å	Ring angle / deg
Phe	zwitterion	0.03	0.64	82.31
Tyr	zwitterion	0.28	1.20	84.92
DOPA	zwitterion	-1.31	1.16	66.19
Trp	zwitterion	-0.68	1.08	61.63
Met	zwitterion	-0.05	-0.02	n/a
Met	cation	0.70	2.81	n/a
Leu	cation	-0.72	1.20	n/a

Geometry optimizations were carried out using the composite density functional method r2SCAN-3c[S7] together with the SMD implicit solvation model for water.[S8] The optimizations were performed in two steps. First, only hydrogen atoms were optimized while all non-hydrogen atoms were kept fixed at their crystallographic positions, in order to obtain chemically reasonable hydrogen-atom positions for ammonium, carboxyl/carboxylate, and water molecules. In the second step, all atoms of the molecular cluster were optimized at the same level.

Counterpoise-corrected interaction energies (Table S41) were then calculated as single-point energies on the optimized geometries using the range-separated hybrid meta-GGA functional ωB97M-V[S9, S10] with the def2-TZVP basis set,[S11] applying tight SCF convergence criteria. Basis-set superposition errors were corrected using the counterpoise procedure of Boys and Bernardi.[S12] For each cluster, the interaction energy was calculated from the energy of the complete complex and the energies of the corresponding fragments evaluated in the full cluster basis using ghost atoms.

The water molecules included in the interaction-energy analysis (Table S41) were assigned according to their positions in the XRD-derived clusters and their hydrogen-bonding roles. Water molecules denoted Wc are located inside the CB7 cavity and form hydrogen bonds with the ammonium region of the guest. Water molecules denoted Wb are bridge waters involved in hydrogen-bonding networks between the amino acid and the CB7 portal region, most often connecting the carboxyl/carboxylate group or the ammonium group with portal carbonyl oxygen atoms. The exact role of each water molecule differs between complexes. For Phe and Tyr, Wc1 is a cavity water molecule stabilizing the ammonium group. In DOPA, Wb1 and Wb2 both bridge the carboxylate group with the CB7 portal region. In Trp, three bridge waters are present: Wb1 is associated with the ammonium/portal region, Wb2 interacts with Wb1, and Wb3 bridges the carboxylate group. In Met3, Wb1 bridges the carboxylate group, whereas Wb2 bridges the ammonium group; these two interactions occur at opposite portals of CB7. In Met4, Wb1 is located close to the rim/cavity region and interacts with the carboxyl group. In both Leu1 and Leu2, Wb1 and Wb2 bridge the ammonium group with the CB7 portal region.

The calculated CB7$\cdots$AA interaction energies show a pronounced dependence on the protonation state of the guest (Table S41). The cationic Met and Leu complexes display much

Table S41: Counterpoise-corrected pairwise interaction energies for selected contacts in XRD-derived CB7–amino acid clusters. Energies are given in kJ mol^{-1}. The number of crystallographic water molecules included in each optimized cluster is indicated in the model name. Here, q_{AA} denotes the formal charge of the amino acid fragment. Wc denotes a cavity water molecule, whereas Wb denotes a bridge water molecule. Dashes indicate that the corresponding water molecule was not present in the selected cluster or that the pair interaction was not evaluated.

Model	q_{AA}	CB7···AA	AA···Wc1	AA···Wb1	AA···Wb2	AA···Wb3
CB7–Phe–2H_2O	0	-157.94	-50.57	-22.09	–	–
CB7–Tyr–2H_2O	0	-171.64	-41.44	-14.02	–	–
CB7–DOPA–2H_2O	0	-200.38	–	-33.67	-34.56	–
CB7–Trp–3H_2O	0	-191.79	–	13.77	-22.26	-29.00
CB7–Met1	0	-120.25	–	–	–	–
CB7–Met2	0	-127.15	–	–	–	–
CB7–Met3–2H_2O	+1	-409.27	–	-66.19	-67.86	–
CB7–Met4–1H_2O	+1	-438.10	–	-64.31	–	–
CB7–Leu1–2H_2O	+1	-401.11	–	-53.29	-28.14	–
CB7–Leu2–2H_2O	+1	-396.92	–	-46.59	-29.40	–

stronger local CB7···AA stabilization than the zwitterionic complexes, consistent with enhanced ion–dipole interactions between the positively charged amino acid and the CB7 carbonyl portal. This local stabilization is consistent with the observation of cationic Met and Leu binding modes in the crystal structures. These pairwise interaction energies should be interpreted as local structural descriptors rather than as direct predictors of solution binding free energies.

To complement the interaction-energy analysis of XRD-derived clusters, representative geometries were selected from the MD-defined hydration states of Phe@CB7 and Tyr@CB7 (Table S42). The snapshots were assigned to hydration states as described in the Molecular Dynamics section, using the common CB7 reference frame defined by the portal centroids and the CB7 mid-plane. For each selected state, a representative frame was chosen to capture the typical arrangement of the guest and nearby water molecules. The resulting counterpoise-corrected energies should be interpreted as local pairwise interaction energies for representative hydration motifs, not as free-energy differences between MD states.

Table S42: Counterpoise-corrected pairwise interaction energies for selected contacts in representative MD-derived hydration states of Phe@CB7 and Tyr@CB7. Energies are given in kJ mol^{-1}. W1 denotes the state-defining water molecule selected for the local interaction analysis: the ammonium-contact cavity water in the *core-contact* state, the non-contact cavity water in the *core-no-contact* state, or the selected portal water in the *portal-only* state. Wb denotes an additional bridge water molecule involved in the local hydration network. $\Sigma E_{\mathrm{local}}$ is the sum of the listed pairwise interaction energies for a given cluster. Dashes indicate that the corresponding water molecule was not present in the selected cluster or that the pair interaction was not evaluated.

Guest	State	CB7–AA	CB7–W1	AA–W1	CB7–Wb	AA–Wb	W1–Wb	$\Sigma E_{\mathrm{local}}$
Phe	*core-contact*	-166.17	-27.06	-43.63	–	–	–	-236.86
	core-no-contact	-174.32	-10.69	-7.43	-33.24	-26.95	1.44	-251.19
	portal-only	-144.24	-26.42	-5.00	-26.23	-37.63	0.44	-239.08
	dry-cavity	-171.07	–	–	-28.40	-33.84	–	-233.31
Tyr	*core-contact*	-152.34	-31.72	-48.52	-33.13	-25.45	-0.86	-292.02
	core-no-contact	-185.68	-30.25	-8.28	-27.30	-36.04	-0.61	-288.16
	portal-only	-171.71	-23.30	-29.70	-27.27	-30.46	0.18	-282.26
	dry-cavity	-186.56	–	–	-33.61	-23.39	–	-243.56

The state-resolved interaction energies show that the direct CB7–AA term and the amino-acid–water terms vary between hydration states. The values are therefore used here as local descriptors of representative hydration motifs, rather than as state free energies.

As an additional local-energy descriptor, force-field interaction-energy components were evaluated for state-assigned MD frames of Phe@CB7 and Tyr@CB7 (Table S43). The analysis compares the redistribution of CB7–AA, AA–W, and CB7–W interactions between the *core-contact* and *portal-only* states.

Table S43: State-resolved force-field interaction-energy components for Phe@CB7 and Tyr@CB7. Energies are reported as mean values in kJ mol^{-1}. For the *core-contact* states, W denotes the ammonium-contact cavity water and the averages were calculated over all frames assigned to this state. For the *portal-only* states, W denotes the selected portal water in representative frames (n = 50); for Tyr this is the OH-associated portal water, whereas for Phe it is the nearest ligand-associated portal water. $n_{\mathrm{W,unique}}$ denotes the number of unique water molecules contributing to the analysis. $E_{\mathrm{local}} = E_{\mathrm{CB7-AA}} + E_{\mathrm{AA-W}} + E_{\mathrm{CB7-W}}$.

Guest	State	n_{frames}	$n_{\mathrm{W,unique}}$	$E_{\mathrm{CB7-AA}}$	$E_{\mathrm{AA-W}}$	$E_{\mathrm{CB7-W}}$	E_{local}
Phe	*core-contact*	11741	58	-168.9	-49.2	-18.1	-236.2
Tyr	*core-contact*	11787	25	-175.1	-49.1	-16.5	-240.6
Phe	*portal-only*	50	49	-170.6	-3.0	-18.4	-192.0
Tyr	*portal-only*	50	50	-184.1	-22.3	-18.9	-225.3

References

[S1] Sheldrick, G. M. *Acta Crystallogr., Sect. A* **2015**, *71*, 3–8.

[S2] Sheldrick, G. M. *Acta Crystallogr., Sect. C* **2015**, *71*, 3–8.

[S3] Wilson, A. J. C.; Geist, V. *Crystal Research and Technology* **1993**, *28*, 110–110.

[S4] Case, D. A. et al. *J. Chem. Inf. Model.* **2025**, *65*, 7835–7843.

[S5] Neese, F. *WIREs Computational Molecular Science* **2012**, *2*, 73–78.

[S6] Neese, F. *WIREs Computational Molecular Science* **2025**, *15*, e70019.

[S7] Grimme, S.; Hansen, A.; Ehlert, S.; Mewes, J. *J. Chem. Phys.* **2021**, *154*, 064103.

[S8] Marenich, A. V.; Cramer, C. J.; Truhlar, D. G. *J. Phys. Chem. B* **2009**, *113*, 6378–6396.

[S9] Vydrov, O. A.; Van Voorhis, T. *J. Chem. Phys.* **2010**, *133*, 244103.

[S10] Hujo, W.; Grimme, S. *J. Chem. Theory Comput.* **2011**, *7*, 3866–3871.

[S11] Weigend, F.; Ahlrichs, R. *Phys. Chem. Chem. Phys.* **2005**, *7*, 3297–3305.

[S12] Boys, S.; Bernardi, F. *Mol. Phys.* **1970**, *19*, 553–566.
